# Binding to RNA regulates Set1 function

**DOI:** 10.1038/celldisc.2017.40

**Published:** 2017-10-24

**Authors:** Pierre Luciano, Jongcheol Jeon, Abdessamad El-kaoutari, Drice Challal, Amandine Bonnet, Mara Barucco, Tito Candelli, Frederic Jourquin, Pascale Lesage, Jaehoon Kim, Domenico Libri, Vincent Géli

**Affiliations:** 1 Marseille Cancer Research Center (CRCM), Aix Marseille University, Institut Paoli-Calmettes. Equipe labellisée Ligue, Marseille, France; 2 Department of Biological Sciences, Korea Advanced Institute of Science and Technology, Daejeon, South Korea; 3 Institut Jacques Monod, Univ Paris Diderot, Sorbonne Paris Cité, CNRS, Bâtiment Buffon, Paris Cedex, France; 4 Université Paris Diderot, Sorbonne Paris Cité, INSERM U944, CNRS UMR 7212, Institut Universitaire d'Hématologie, Hôpital St. Louis, Paris, France

**Keywords:** Set1, transcription, RNA binding, H3K4 methylation

## Abstract

The Set1 family of histone H3 lysine 4 (H3K4) methyltransferases is highly conserved from yeast to human. Here we show that the Set1 complex (Set1C) directly binds RNA *in vitro* through the regions that comprise the double RNA recognition motifs (dRRM) and N-SET domain within Set1 and its subunit Spp1. To investigate the functional relevance of RNA binding, we performed UV RNA crosslinking (CRAC) for Set1 and RNA polymerase II in parallel with ChIP-seq experiments. Set1 binds nascent transcripts through its dRRM. RNA binding is important to define the appropriate topology of Set1C distribution along transcription units and correlates with the efficient deposition of the H3K4me3 mark. In addition, we uncovered that Set1 binds to different classes of RNAs to levels that largely exceed the levels of binding to the general population of transcripts, suggesting the Set1 persists on these RNAs after transcription. This class includes RNAs derived from *SET1*, Ty1 retrotransposons, specific transcription factors genes and snRNAs (small nuclear RNAs). We propose that Set1 modulates adaptive responses, as exemplified by the post-transcriptional inhibition of Ty1 retrotransposition.

## Introduction

Highly conserved histone proteins undergo several types of covalent modifications including acetylation, methylation, phosphorylation, ubiquitylation, SUMOylation, citrullination and ADP-ribosylation [1]. These modifications that are deposited and removed by specific chromatin-modifying enzymes can either directly alter the chromatin architecture or create docking sites that facilitate the binding of specific domains present in chromatin readers [2]. These readers in turn recruit chromatin remodeling enzymes or additional chromatin modifiers to shape chromatin landscapes that regulate DNA accessibility [3]. Among these marks, methylation of lysine 4 on histone H3 (H3K4) has aroused considerable interest [4]. In mammals, this modification is catalyzed by at least six different complexes that differ by their catalytic SET domain subunit (Set1a, Set1b, Mll1, Mll2, Mll3 and Mll4) [5] but share a protein module comprises WDR5, RbBP5, ASH2L and DPY-30, which binds to the catalytic SET domain and stimulate H3K4 methyltransferase activity [6]. Each complex contains additional factors specifying their recruitment to chromatin and their biological effect [7].

In *Saccharomyces cerevisiae*, all H3K4 methylation is carried out by a complex called COMPASS (for complex of proteins associated with Set1) or Set1C (for Set1 Complex) [8]. The catalytic subunit Set1 acts as a scaffold for seven other components (Swd1 (mammalian homolog RbBP5), Swd2 (WDR82), Swd3 (WDR5), Bre2 (ASHL2), Sdc1 (DPY-30), Spp1 (CFP1) and Shg1 (BOD1)) [9]. Swd1, Swd3, Bre2 and Sdc1 associate with the SET domain of Set1 to form the SETc that is minimally sufficient to methylate free H3 *in vitro* [9], whereas Spp1 and Shg1 directly associate to Set1 by binding to the N-SET domain and the Set1 central region, respectively [10]. The loss of individual Set1C subunits differentially affects Set1 stability, complex integrity, the pattern of global H3K4 methylation and the distribution of H3K4 methylation marks along active genes [11]. The WD40 repeat protein Swd2 is the only essential subunit of Set1C and its depletion strongly affects Set1 stability and H3K4 methylation [9]. Swd2 also belongs to the APT complex (for ‘*associated with Pta1*’), which is part of the cleavage polyadenylation factor [12]. Several studies suggested a functional link between Set1C and 3′-end formation/termination [13] but it remains unknown how the binding of Swd2 to either of the two complexes (Set1C and APT complex) is regulated. Other regions outside of the SET domain have been reported to regulate Set1 catalytic activity, including the N-SET domain, the double RNA recognition motif (dRRM) and a centrally located auto-inhibitory domain, but the mechanism underlying such regulation is still elusive [14].

Genome-wide studies in yeast indicate that active transcription is characteristically accompanied by histone H3K4 trimethylation (H3K4me3) at the 5′-end of genes and by H3K4 di- and monomethylation (H3K4me2 and H3K4me1) at downstream nucleosomes [15]. H3K4me3 can also be found at the 3′-end of a number of genes most likely reflecting the presence of antisense ncRNAs (non-coding RNAs) [16]. These H3K4 methylation patterns correlate with Set1 occupancy that is higher at the 5′-end of coding regions of highly transcribed RNA polymerase II (RNAPII) genes and decreases at more distal nucleosomes [17]. Set1 has been reported to associate with the elongation complex in the early stages of the transcription cycle, which is thought to contribute to the prevalence of H3K4me3 at the 5′-end of active genes. Recruitment occurs when the carboxyl terminal domain of RNAPII is preferentially phosphorylated at the serine in the fifth position (Ser5) of its heptad repeats, which has been reported to depend on the Paf1 complex [18]. However, direct interactions that underpin the recruitment of Set1C to actively transcribed genes remain to be characterized. Although interaction of Set1C with chromatin was proposed to be mediated by the interaction of Swd2 with ubiquitylated H2B (H2Bub) [19], this model has been challenged by *in vitro* reconstitution experiments showing that the Swd2-deficient Set1C can methylate chromatinized H3K4 in an H2B ubiquitylation-dependent manner [20]. Thus, current models to explain Set1 recruitment and the establishment of H3K4 methylation along genes *in vivo* still need to be improved.

Set1 contains two tandem RRMs, RRM1 and RMM2 (dRRM) [14]. We previously reported that Set1 RRM1 contains the canonical RRM-fold but lacks some typical RNA-binding features. Consistently, RRM1 is necessary but not sufficient for Set1 to bind RNA *in vitro* and RRM2 was also shown to be required [21]. Deletion or mutation of RRM1 has been shown to lead to decreased H3K4me3 in the 5′ regions of active genes along with an increase in H3K4me2 [14], opening the possibility that a potential RNA-binding activity of Set1 could regulate Set1 occupancy and/or the distribution of H3K4 methylation [22].

Here, we show that Set1 binds directly RNA and that its dRRM and N-SET, as well as Spp1, contribute to Set1 RNA binding *in vitro* in the context of a reconstituted Set1C. By combining ChIP-seq and CRAC experiments of Set1 and Set1 mutants that have lost the ability to bind RNA, we show that Set1 RNA-binding activity mediated by its dRRM does not affect Set1 recruitment to chromatin per se but maintains Set1 in the 5′ region of genes. We propose that RNA binding to Set1 increases the time of residency of Set1C in the proximity of chromatin allowing additional time for H3K4 trimethylation in the 5′-end of genes. Our results also indicate that Set1 strongly associates, presumably post-transcriptionally to transcripts produced by specific classes of genes, including snRNAs small nuclear RNAs, Ty1 and adaptive response genes. In particular, we show that Ty1 retrotransposition is negatively regulated by Set1 at a post-transcriptional level.

## Results

### Binding of RNA* in vitro* by reconstituted Set1C involves the dRRM and N-SET domains of Set1

**Figure 1:**
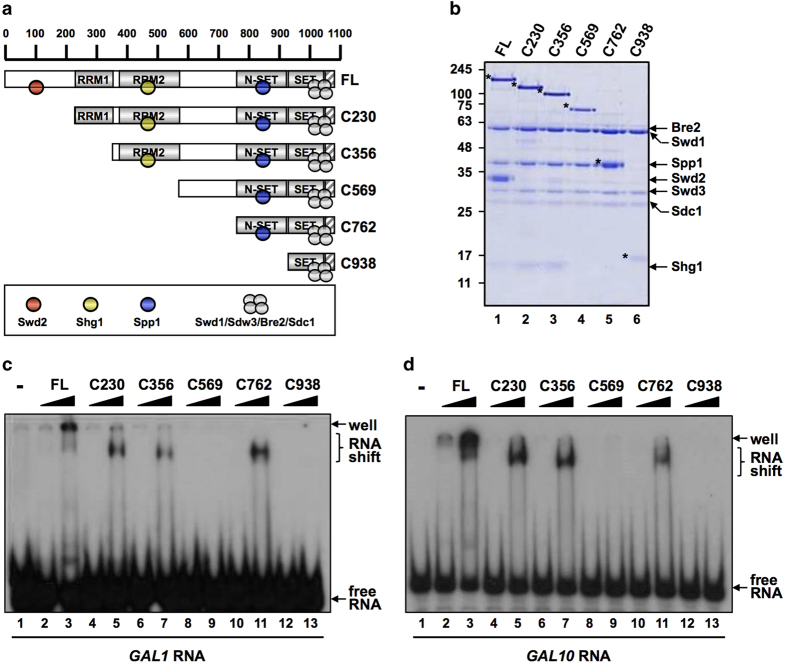
Purified Set1C binds directly to RNA *in vitro.* (**a**) A schematic diagram of Set1 and derived fragments with predicted RRM1, RRM2, N-SET and post-SET (hatched box) domains and associated subunits. FL indicates full-length. (**b**) Sodium dodecyl sulfate–polyacrylamide gel electrophoresis (SDS–PAGE) and Coomassie blue staining of purified Set1Cs reconstituted with baculoviruses expressing FLAG-Set1 or FLAG-Set1 fragments and untagged subunits. FLAG-Set1 polypeptides are marked by asterisks. (**c,** **d**) Radiolabeled GAL1 (**c**) and GAL10 (**d**) transcripts were subjected to *in vitro* RNA electrophoretic mobility shift assays with 0.5 (lanes 2, 4, 6, 8, 10 and 12) or 2.5 (lanes 3, 5, 7, 9, 11 and 13) pmoles of indicated Set1Cs.

Our previous results suggested that purified Set1 RRM1-RRM2 (dRRM) binds RNA *in vitro *[21]. We reconstituted the whole Set1C [20] to analyze Set1 RNA binding in the context of the complex form of Set1 with its associated subunits. Set1C was purified from insect cells expressing FLAG-fused full-length or truncated Set1 together with the seven other subunits (Figure 1a and b). Purified complexes lacking specific domains of Set1 were incubated with *in vitro* transcribed and purified *GAL1* and *GAL10* mRNAs. Interaction was probed by electrophoretic mobility shift assay (Figure 1c and d). We found that reconstituted Set1C was able to directly bind *GAL1* and *GAL10* mRNAs *in vitro*. In agreement with our previous results, a truncated Set1 lacking the two RRM motifs (C569) was unable to bind RNA. Surprisingly, we found that the C762 fragment encompassing the N-SET and the SET domains (Figure 1a) was able to bind RNA in contrast to C569 and C938 that could not. These results suggest that N-SET contributes to Set1 RNA binding, which might be inhibited by the region between residues 569 and 762 (Figure 1c and d).

**Figure 2:**
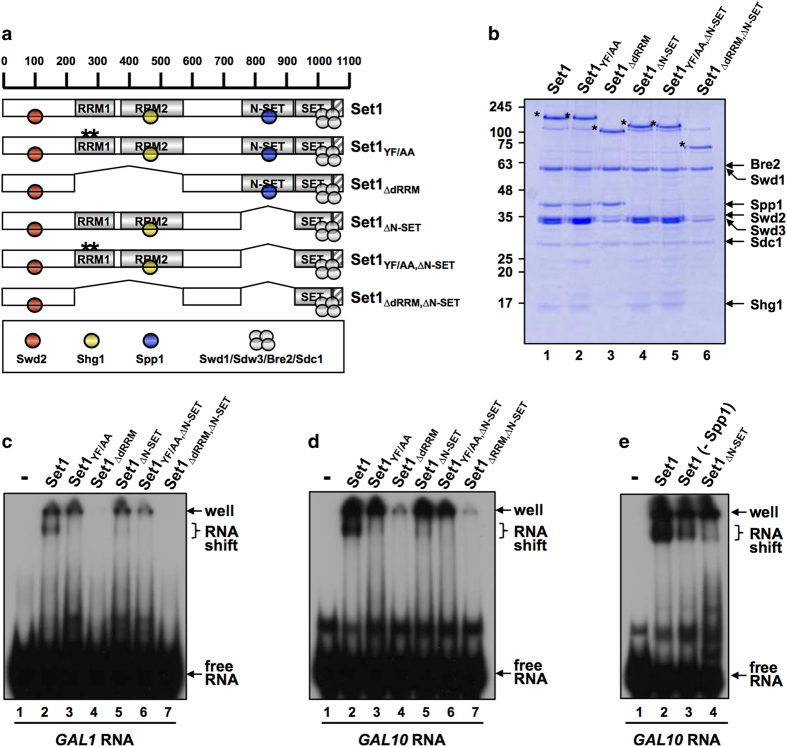
Set1C RNA binding requires dRRM, the N-SET domain, and Spp1. (**a**) A schematic representation of Set1, Set1_YF/AA_ and Set1 deletion mutants with predicted RRM1, RRM2, N-SET and post-SET (hatched box) domains and associated subunits. (**b**) Sodium dodecyl sulfate–polyacrylamide gel electrophoresis (SDS–PAGE) and Coomassie blue staining of purified Set1Cs reconstituted with baculoviruses expressing FLAG-Set1 or FLAG-Set1 fragments and untagged subunits. (**c**, **d**) Radiolabeled *GAL1* (**c**) and *GAL10* (**d**) transcripts were subjected to *in vitro* RNA electrophoretic mobility shift assays with indicated Set1Cs. (**e**) Binding of Set1C and Set1C lacking Spp1 to radiolabeled GAL10. Set1C containing Set1_∆N-SET_ is also shown.

To further confirm these results, we introduced in the full-length Set1 the Y_271_F_272_/AA mutation previously shown to decrease the RNA-binding activity of dRRM *in vitro *[21], as well as a deletion of the dRRM, or the N-SET domain, and a combination of these mutations [21] (Figure 2a and b). Consistent with Figure 1, either the Y_271_F_272_/AA mutation or dRRM deletion strongly affected Set1C RNA-binding *in vitro* (Figure 2c and d). Deleting only the N-SET domain also significantly decreased RNA binding suggesting that the N-SET domain is important for RNA binding (Figure 2c and d). However, we did not detect interaction of the N-SET domain alone with RNA (data not shown) indicating that the N-SET domain is not sufficient to bind RNA. As expected, combining alterations of the dRRM with the N-SET deletion abolished Set1 RNA-binding activity *in vitro*. We next assessed whether Set1C subunits contribute by themselves to the RNA-binding activity of Set1C by monitoring the RNA-binding activity of each subunit. None of the Set1C subunits were found to bind *GAL10* mRNA (Supplementary Figure S1A and B). Finally, as the N-SET domain binds Spp1, we asked whether only omitting Spp1 in Set1C reconstitution also affected RNA-binding activity. The results shown in Figure 2e indicated that Spp1 was required for Set1C to bind RNA despite the fact that Spp1 by itself does not bind RNA.

Collectively, these results show that the fully reconstituted Set1C has the ability to bind mRNAs *in vitro*. Unexpectedly, not only the dRRM motif but also the N-SET domain and Spp1 contributed to the ability of Set1 to bind RNA. Therefore, Set1C RNA binding requires the presence of multiple protein surfaces comprising the dRRM, as well as N-SET domain and Spp1.

### Altering Set1 dRRM affects Set1 distribution along genes

Before addressing Set1 binding to RNA *in vivo,* we performed ChIP-seq analysis to determine the genome-wide occupancy of Set1 and Set1 mutants. ChIP-seq experiments were carried out from *set1∆* cells expressing N-terminal tagged (Z-tag-Tev-6HIS) version of Set1 (PTH-Set1) [23]. Because in cells grown in SC-LEU-TRP medium, the amount of PTH-Set1 was similar to that of endogenous Set1 (Supplementary Figure S2A) we used these conditions for ChIP-seq and all subsequent experiments.

To address the importance of the Set1 domains involved in RNA-binding *in vitro* for chromatin binding, we performed ChIP-seq experiments of PTH-Set1 and of its mutant forms (set1_YF/AA_, set1_∆dRRM_, set1_∆N-SET_ and set1_∆dRRM,∆N-SET_) with an anti-Set1 mouse monoclonal antibody (anti-Set1 mAb) [24] that recognizes a Set1 epitope between residues 700 and 761 (Supplementary Figure S2B and C). In parallel, we also performed ChIP-seq of a Myc-Set1 strain with an anti-Myc antibody (9E10). The occupancy profiles for PTH-Set1 and Myc-Set1 at selected genes were overall similar and both datasets were highly correlated (Supplementary Figure S3A, B and C). Set1 occupancy was maximum beyond the H3K4me3 peak and upstream of the H3K4me2 peak and was slightly 3′-shifted relative to RNAPII occupancy (Supplementary Figure S3D), in agreement with previous results indicating that Set1 and its subunits are recruited at the 5′ region of active genes transcribed by RNAPII [17].

**Figure 3:**
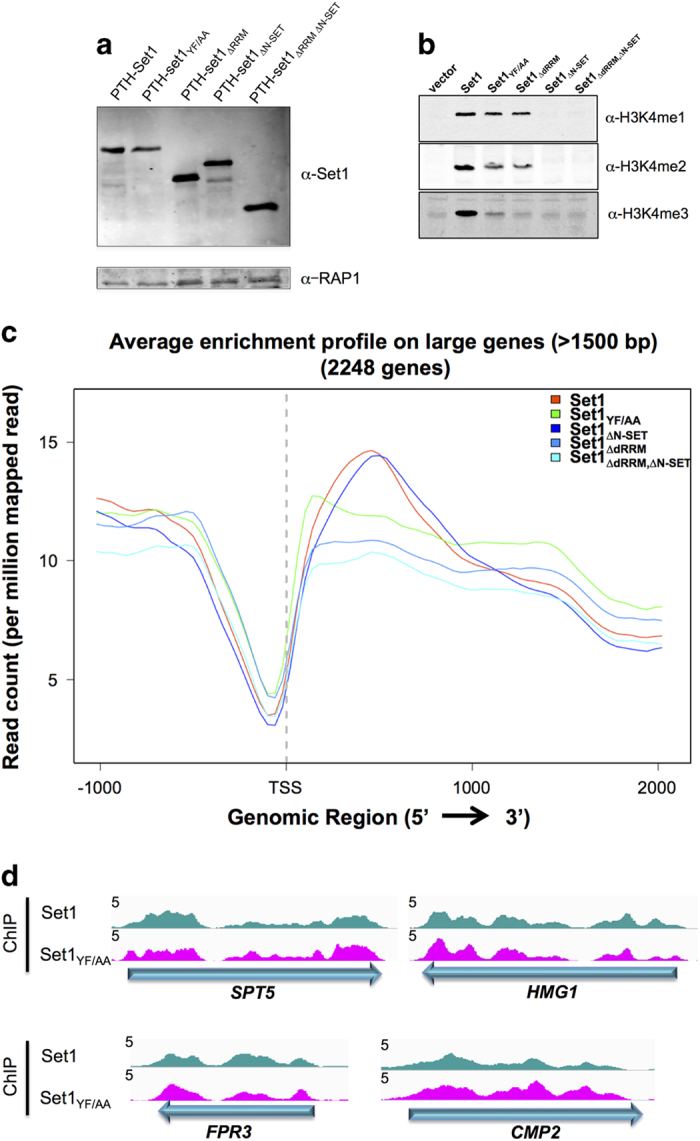
RRM but not N-SET regulates the genome-wide occupancy of Set1. (**a**, **b**) W303 *set1*∆*::TRP1* pRS415-nHTP-*SET1* (and *SET1* mutant forms) cells were grown in SC -TRP-LEU. Protein levels of Set1 (**a**) and methylated histone H3 (**b**) were verified by western blots using anti-Set1 mAb and anti-H3K4me1, me2, and me3 antibodies, respectively. A Rap1 loading control is shown. (**c**) Average enrichment profiles of PTH-Set1 and PTH-Set1 mutants in genes>1500 bp. Read counts were normalized to read counts per million of mapped reads. ChIP-seq experiments were performed with the anti-Set1 mAb from *set1*∆*::TRP1* cells expressing PTH-Set1 and PTH-Set1 mutants from the pRS415-nHTP (grown in SD -TRP -LEU). (**d**) Normalized occupancy profiles of Set1 at the indicated genes. Graphs were normalized to 10 million mapped reads for each mutant.

Set1 protein amount was controlled in the different mutant strains by western blot (Figure 3a). We reproducibly observed a reduction of the PTH-Set1_YF/AA_ amount by about 1.3–1.5-fold in all experiments [21], whereas deleting dRRM increased the stability of Set1. PTH-Set1 was functional, as it supported wild-type levels of H3K4me3 when expressed in a *set1∆* background. In contrast, H3K4me3 was globally abolished when the dRRM and N-SET functions were compromised (Figure 3b). To evaluate the importance of the Set1 domains characterized *in vitro*, we next assessed the occupancy of PTH-Set1 and mutant forms by ChIP-seq experiments.

Surprisingly, deleting dRRM or mutating it in PTH-Set1_YF/AA_ did not affect the distribution of the individual ChIP signals calculated *per* each mRNA-coding gene (Supplementary Figure S4 for PTH-Set1_YF/AA_, and data not shown). To analyze the chromatin distribution of Set1 and its mutant derivatives in more details, we generated normalized occupancy profiles over large genes, which allows a better spatial resolution of recruitment regions. As shown in Figure 3c, the aggregate signal for Set1_YF/AA_ and Set1_∆dRRM_ shows reduced occupancy in the 5′ region of genes relative to wild-type (WT) Set1, which is compensated by an average increase in the 3′-end of genes to generate the observed unchanged overall signal on a *per gene* basis. This trend is illustrated in Figure 3d for individual genes. Deletion of the N-SET domain had no detectable impact in the distribution of signals (data not shown) or the average profile of the signal (Figure 3c).

These results, together with the strong impact of the Set1_YF/AA_ mutation on *in vivo* RNA binding described below, indicate that the dRRM domain of Set1 is not required for the overall recruitment of Set1 to chromatin, but is essential for its normal distribution along genes. Importantly, the N-SET domain that is essential for Set1C catalytic activity (Figure 3b) and is required to bind RNA *in vitro,* is not involved in the recruitment and positioning of Set1.

### Set1 binding to RNA *in vivo* is determined by both co-transcriptional and post-transcriptional components

We next used the CRAC procedure to analyze *in vivo* the genome-wide RNA binding of Set1 and Set1_YF/AA_ whose RNA-binding activity is compromised *in vitro* to detect *in vivo* RNA-protein interactions [25]. Briefly, tagged RNA-binding proteins are UV crosslinked to their targets *in vivo* and purified by three sequential steps of affinity selection, two of which are under denaturing conditions. The associated RNA is isolated and sequenced. We used the same PTH-Set1 and PTH-*set1*_*YF/AA*_ constructs and growth conditions used for the ChIP-seq. A non-crosslinked sample was processed in parallel as a control for specificity. A spike in control was generated by adding to the *S. cerevisiae* cultures 0.5% of *S. pombe* cells expressing a non-relevant HTP-tagged protein that binds RNA and that was purified with *S. cerevisiae* Set1. The number of reads mapping to the *S. pombe* genome was used for normalization. We also monitored RNAPII distribution by the same CRAC technique, which provides high-resolution information about the level of transcription.

**Figure 4:**
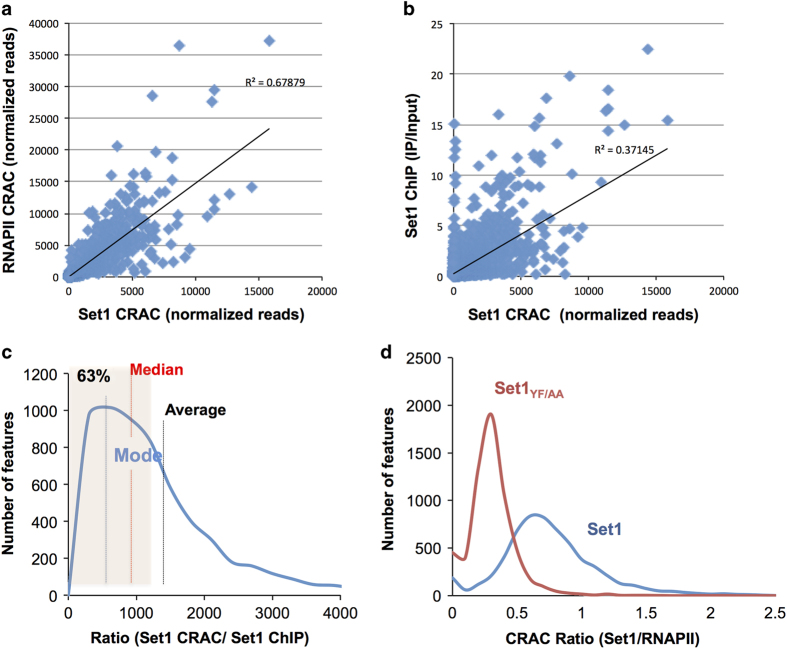
Set1 binding to RNA occurs via two different modes and requires its dRRM. (**a**) Dispersion plot showing high correlation between the Set1 and RNAPII CRAC signals. The determination coefficient (*r*^2^) of the linear, least squares regression is indicated. (**b**) Dispersion plot of Set1 CRAC signals versus Set1 ChIP-Seq (IP/input). (**c**) Distribution of the Set1 CRAC/Set1 ChIP ratios for all mRNAs coding genes. The average, mode and median are indicated to highlight the asymmetry of the distribution. The shaded area corresponds to the percentage of features whose Set1 CRAC/ChIP ratios are included in a range symmetrically positioned around the mode. (**d**) Distribution of the Set1/RNAPII CRAC ratios in cells expressing PTH-Set1 and PTH-Set1_YF/AA._

We identified 2543 mRNAs displaying high-confidence Set1 RNA crosslinking sites for which the number of reads obtained for the crosslinked sample (Set1 CL) was >5-fold over the no-crosslinked sample (Set1 No-CL) (Supplementary Table S1). Set1 was found to bind mRNAs but also several other transcript classes (Supplementary Figure S5). Set1 binds at least partially during transcription, as witnessed by the significant representation of intronic RNAs in the crosslinked material (see below). For assessing to what extent Set1 binds the RNA during transcription, we sought correlations between the Set1 CRAC signal and the levels of RNAPII occupancy as determined by RNAPII CRAC (Figure 4a) for all mRNA-coding genes. We also compared the Set1 CRAC signal with Set1 recruitment to chromatin as determined by ChIP-Seq (Figure 4b). We reasoned that if binding to the RNA were co-transcriptional, theses datasets should be highly correlated. Consistent with this notion, binding of Set1 to the RNA correlated remarkably well both with RNAPII CRAC (Figure 4a, *r*^2^=0.68; *P*=3*E*^-22^) and recruitment of Set1 to chromatin as measured by ChIP (Figure 4b, *r*^2^=0.37; *P*=9*E*^-10^).

The distribution of Set1 CRAC/ChIP ratios was clearly not symmetric as could have been expected for a homogeneous population with random variability (Figure 4c). Rather, it was markedly skewed toward high values (compare the difference between the mode, the median and the average in Figure 4c) with only 63% of the population symmetrically distributed around the mode (shaded area) and the remaining values tailing over a wide range of higher ratios. This suggests the existence of at least two classes of genes: one major, for which the levels of RNA binding relative to chromatin-associated Set1 are relatively homogeneous; the second displaying levels of Set1 binding to RNA that are generally higher than expected based on the sole co-transcriptional interaction. Overall, these analyses strongly suggest that the levels of Set1 binding to the RNA detected by CRAC are generally dominated by a co-transcriptional component but also contain a post-transcriptional component that might predominate for some genes (Supplementary Table S1). Snapshots of the second class of mRNA are shown in Supplementary Figure S6A and B. Interestingly, the feature with the highest Set1 crosslinking signal was the *SET1* mRNA, which is fully consistent with the notion that it associates with the Set1C containing Set1, Swd1, Spp1 and Shg1 during its co-translational assembly [26]. We further sought to determine whether genes encoding this specific class of transcripts (Supplementary Table S1) have physical and/or functional associations. Evidence that many of the proteins encoded by these genes are linked in reliable networks stemmed from computational analysis [27]. Gene ontology analysis revealed that genes whose transcripts were strongly bound by Set1 included some involved in chromosome segregation and many transcription factors (DNA-binding proteins) involved in adaptive responses (Supplementary Figure S6C).

**Figure 5:**
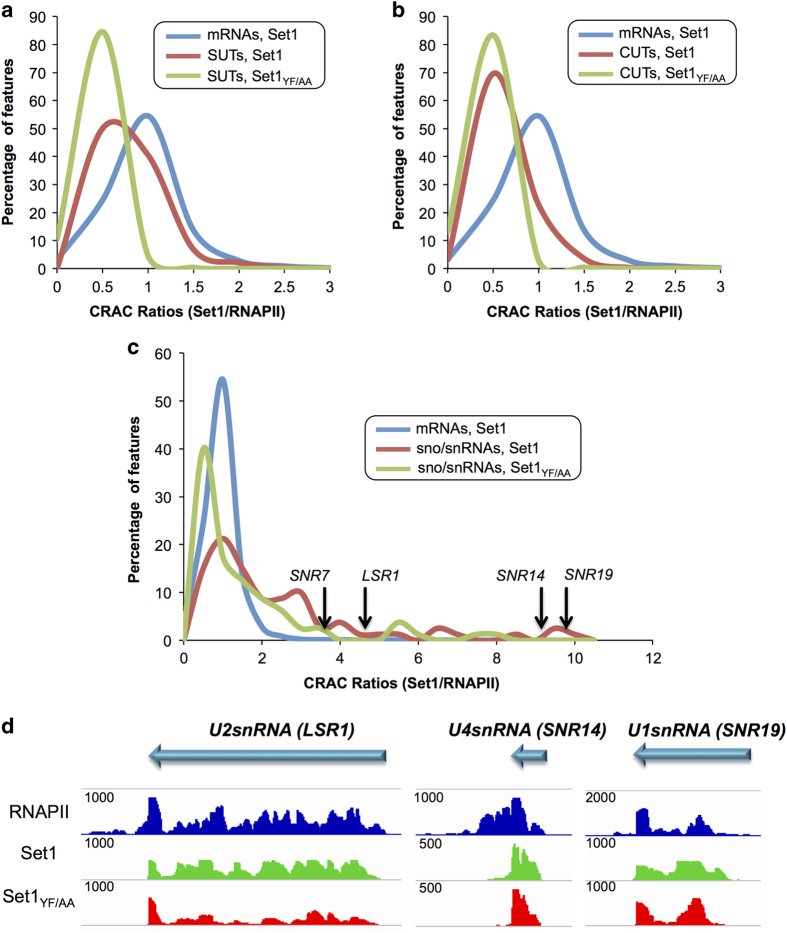
Set1 binds to different classes of RNA. (**a**, **b**, **c**) Normalized distribution of Set1/RNAPII CRAC ratios in cells expressing PTH-Set1 and PTH-Set1_YF/AA_ for the indicated classes of RNA. The position in the distribution of each RNAPII-transcribed snRNAs is indicated in C. The distribution of ratios for mRNAs (Figure 4d) is shown in each graph. (**d**) Snapshots for Set1, Set1_YF/AA_ and PolI CRAC normalized signals for the indicated spliceosomal RNAs.

Mutation of the RNA-binding domain in Set1_YF/AA_ led to a marked decrease in the Set1 CRAC signal, which affected uniformly the whole population of Set1 targets as indicated by a general shift in the distribution of Set1/RNAPII CRAC ratios in the *set1*_*YF/AA*_ mutant relative to WT (Figure 4d). These data demonstrate that the YF/AA mutation in Set1 dramatically affect the interaction of Set1 with mRNAs *in vivo*, although this interaction was not totally abolished but partially maintained with a different topology (see below). Set1 was found to bind with similar *set1*_*YF/AA*_ dependency stable unannotated transcripts, cryptic unstable transcripts and sno/snRNAs (Figure 5a–c). The first two classes interact with Set1 to a somewhat lower extent even when normalized to the RNAPII CRAC signal, maybe because these RNAs are unstable and the post-transcriptional component might contribute to less to the CRAC signal. Many sno- and snRNAs appear to be bound by Set1 post-transcriptionally, as suggested by the large distribution of Set1/RNAPII CRAC values (Figure 5c) and indicated by the general lack of signal in the regions of the precursor (Figure 5d for the U1, U2 and U4 snRNAs and data not shown). Interestingly, spliceosomal snRNAs were among the strongest binders (Figure 5d), possibly suggesting a role of Set1 in splicing.

### Aggregated distribution of Set1 binding on RNAs

**Figure 6:**
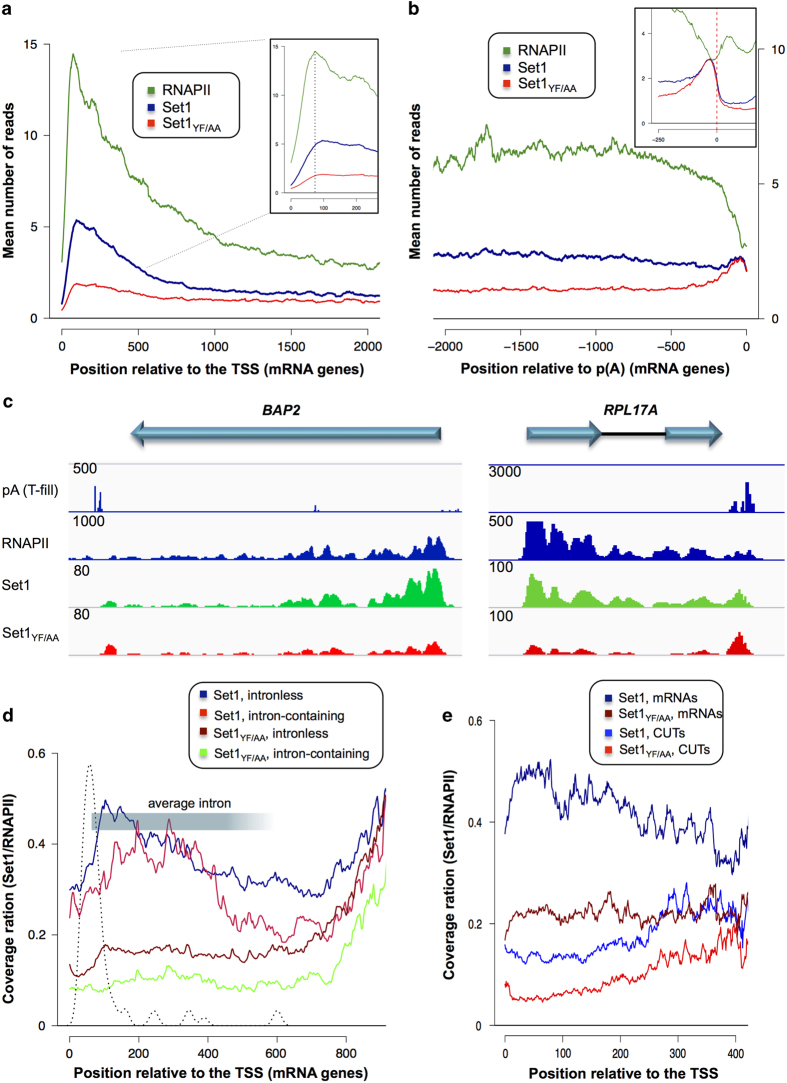
Distribution of Set1-binding sites on RNA. (**a**, **b**) Metagene analysis of Set1 and Set1_YF/AA_ RNA-binding signals on mRNAs as observed by CRAC compared with polymerase occupancy (**a**) features aligned on transcription start site; (**b**) features aligned on wPAS. Insets contain zooms of relevant regions. (**c**) Snapshots for Set1, Set1_YF/AA_, and PolI CRAC normalized signals at the 5′ region of *BAP2* and *RPL17-A* mRNAs. (**d,** **e**) Metagene analysis of Set1 and Set1_YF/AA_ RNA-binding signals, (**d**) on intronless and intron-containing genes, the distribution of intron position is indicated as well as the average position of introns; (**e**) to cryptic unstable transcripts (CUTs) compared with size matched open reading frame (ORF).

We profiled the distribution of RNA-associated Set1 for different features aligned on the transcription start site. As co-transcriptional RNA binding at any given position is likely to be strongly dependent on the level of transcription, we plotted in parallel RNAPII occupancy as defined by the CRAC signal. As shown in Figure 6a, binding of Set1 to the RNA was slightly delayed relative to the appearance of the RNAPII signal (see inset in Figure 6a), which resulted in a relative Set1/RNAPII signal building up in the first 100–250 nt of transcription. This is consistent with the notion that Set1 is recruited co-transcriptionally to the RNA and suggests that it binds the nascent transcript after interacting directly with the polymerase. In the *set1*_*YF/AA*_ mutant, the CRAC signal was markedly reduced over most of the length of the transcription unit, particularly in the 5′ region.

To assess the distribution of the Set1 signal in the 3′ region of genes we first calculated a positionally weighted average p(A) site (wPAS) for every gene (see Materials and Methods section). This was necessary to improve the quality of the alignment in the 3′-end of genes as most genes have multiple polyadenylation sites. To this end, we used T-fill data [28] and assigned to every p(A) addition site a weight depending on the intensity of the signal at that position. This was used to generate a positionally weighted average p(A) site (wPAS). As shown in Figure 6b, the polymerase signal declines in this region, either because of multiple sites of termination or to increased speed. Interestingly, the Set1 signal is maintained and actually slightly increases immediately before the wPAS. Intriguingly, this increase is maintained in the YF/AA mutant, to the point that the signals for the mutant and WT Set1 are identical in this region. This surprising observation indicates that the YF/AA mutation does not affect the binding to the RNA in this region of the transcripts. Whether this 3′ peak is mainly because of the co-transcriptional or post-transcriptional binding of Set1 to the RNA cannot be determined from these experiments. Note that if the 3′ peak were formed co-transcriptionally, its intensity relative to the polymerase signal, which decreases in this region (Figure 6b), would be higher than in other regions of the RNA (see Discussion). After the wPAS, both the WT and mutant Set1 signals decrease steadily (Figure 6b, inset), indicating that they are significantly above background within the range of the transcription unit. The presence of a Set1 3′ peak can be readily observed at individual genes, most prominently in the mutant for which the signal before the peak is generally lower (Figure 6c; see also snapshots for the *SET1, SLK19,* and *SWI1* loci in Supplementary Figure S6), indicating that this behavior is not limited to a small set of genes. These data are compatible both with increased co-transcriptional recruitment of Set1 in the region of termination and with a post-transcriptional binding to the mRNA in the immediate vicinity of the poly(A) site. Importantly and surprisingly, in both cases the interaction with the RNA is not dependent on dRRM.

Prompted by the strong binding of Set1 to snRNAs, we assessed the profile of Set1 binding to intron-containing RNAs by comparing it with size matched mRNA-coding genes. As shown in Figure 6d, Set1 bound intronic transcripts with similar or even better efficiency than non-intronic RNAs, causing a slight downstream shift of the 5′-peak of Set1 binding. Binding of the Set1_YF/AA_ was similarly affected at intron-containing genes, as well as to the general population. We also assessed binding to cryptic unstable transcripts, a class of transcripts that are unstable in WT yeast because they are rapidly degraded in the nucleus [29], which we compared with matched size small open reading frames. Set1 binding to these features was lower than at small open reading frames, even when normalization to RNAPII was applied (Figure 6e) to account for the generally different levels of transcription. This could be due either to specificities residing in the sequence of the cryptic unstable transcript, or to nuclear degradation of these RNAs.

### Set1 represses Ty1 retrotransposition post-transcriptionally

**Figure 7:**
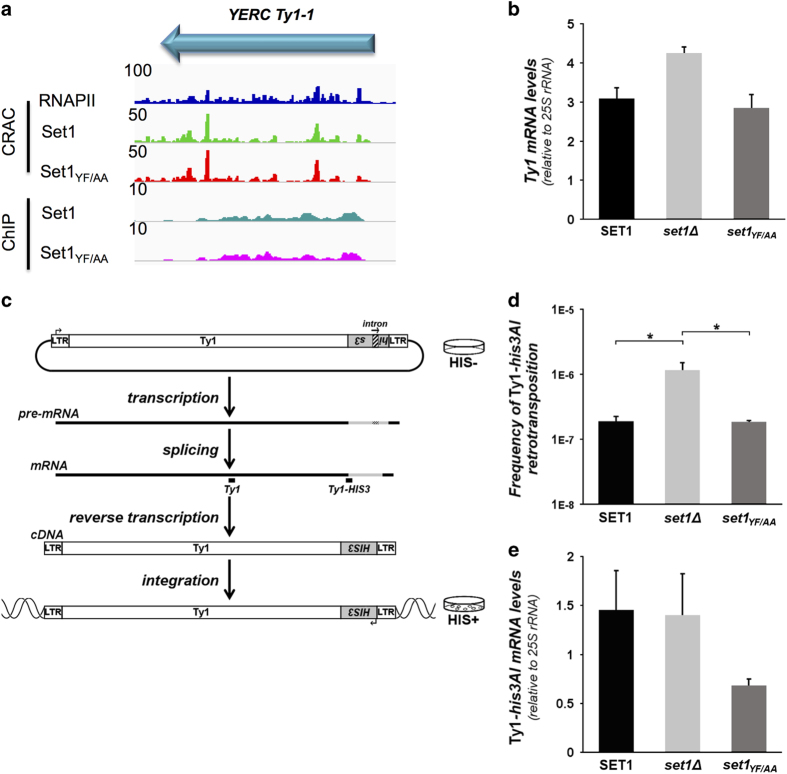
Set1 represses Ty1 retrotransposition post-transcriptionally. (**a**) Example of the Set1 and Set1_YF-AA_ binding on Ty1-1 mRNA. The Set1 CRAC and ChIP profiles are shown. (**b**) Global expression of endogenous Ty1 retrotransposons in *SET1, set1Δ* or *set1-YF/AA* yeast cells, monitored by quantifying Ty1 mRNA levels by RT-qPCR (normalized to 25 S rRNA values; mean±s.d.; *n*=3). (**c**) Ty1 retrotransposition assay from a plasmid expressing a Ty1 element tagged with the *his3AI* reporter gene [33]. An intron is inserted in the *HIS3* gene in an antisense orientation in a spliceable orientation in the Ty1 transcript resulting in a Ty1 complementary DNA (cDNA) bearing a functional *HIS3* gene. The cDNA can then be integrated into the host genome. Cells that sustain a Ty1-*HIS3* retrotransposition event give rise to His+ colonies [32]. The position of the qPCR amplicons used to amplify all the Ty1 mRNAs in (**b**) and the Ty1 reporter mRNA specifically expressed from the plasmid in (**e**) are indicated (*Ty1* and *Ty1-HIS3* amplicons, respectively). (**d**) Frequency of Ty1-*his3AI* retrotransposition in *SET1, set1Δ* or *set1-YF/AA* yeast cells (number of His+ prototrophs divided by the total number of cells; mean±s.d.; *n*=3). *p≤0.05 (Welch’s *t*-test). (**e**) Plasmid Ty1-*his3AI* expression in *SET1, set1Δ* or *set1-YF/AA* yeast cells monitored by quantifying Ty1-HIS3 mRNA levels by RT-qPCR (normalized to 25S rRNA values; mean±s.d.; *n*=3).

Among the mRNAs that were strongly bound by Set1, presumably post-transcriptionally, we also found Ty1 retrotransposon (Figure 7a). Binding of Set1 to Ty1 mRNA was not affected by the YF/AA mutation suggesting that Set1 binding to Ty mRNA does not involve its dRRM (Figure 7a). The Set1_YF/AA_ mutation had no major effect on steady-state Ty1 mRNA levels (Figure 7b) as previously reported for the *set1∆* mutant [30,31]. This indicates that Set1 binding does not affect Ty1 mRNA expression or stability. To assess whether Set1 affects Ty1 retrotransposition, we performed a typical retrotransposition assay based on a Ty1 element marked with a *his3AI* reporter gene on a plasmid, which confers His^+^ prototrophy to cells upon retrotransposition (Figure 7c). In the absence of Set1, the frequency of Ty1 retrotransposition significantly increased (Figure 7d), whereas no change in Ty1*HIS3* mRNA levels was observed (Figure 7e). This indicates that Set1 can repress Ty1 mobility at a post-transcriptional stage. In contrast, Set1-YF/AA, which retains the ability to bind Ty1 mRNAs, repressed Ty1 retrotransposition as efficiently as WT Set1. These results suggest that Set1 binding to Ty1 mRNA could impair Ty1 mRNA export, translation or encapsidation, all essential steps to Ty1 retrotransposition efficiency. Of note, the less than twofold decrease in Ty1-*his3AI* mRNA levels observed in the *set1-YF/AA* mutant may not affect Ty1 retrotransposition (Figure 7e), as much more Ty1 mRNAs are produced than effective transposition events occurring in cells [32]. However, we cannot exclude that the slight defect in Ty1-*his3AI* mRNA levels may mask a slight increase in Ty1 cDNA integration that could be facilitated by the modification of the histone methylation status of the yeast genome in the *set1-YF/AA* mutant.

### Reduced H3K4me3 levels are due to defective recruitment or positioning of Set1 during transcription

**Figure 8:**
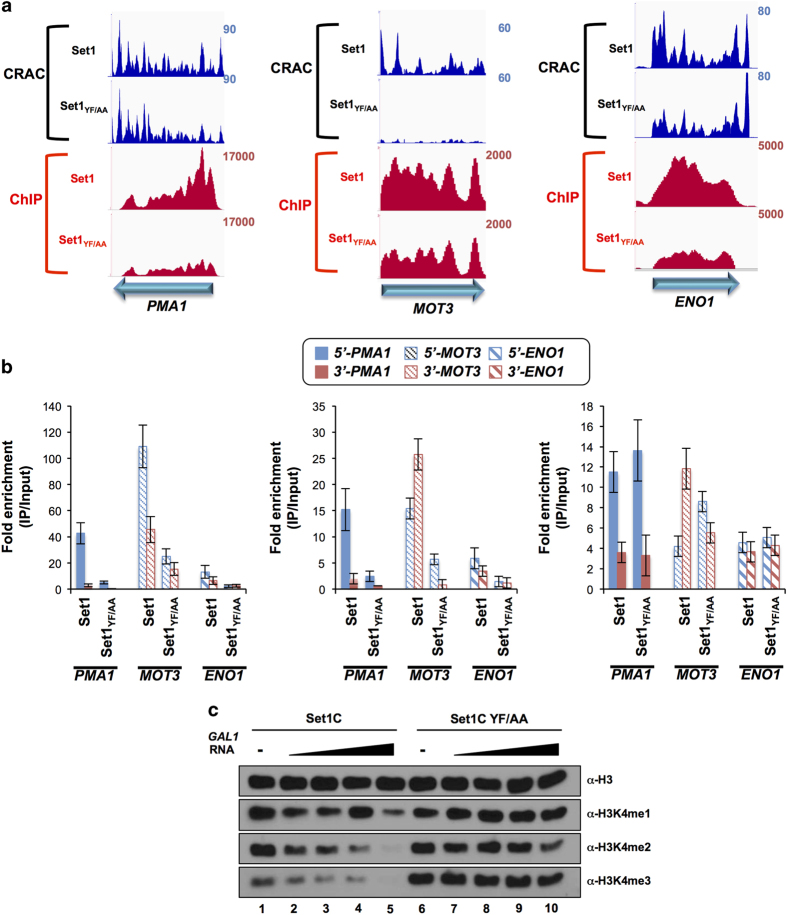
Reduction of Set1 occupancy and RNA-binding activity in Set1_YF/AA_ correlates with reduced H3K4me3 levels. (**a**) ChIP-seq and CRAC signals of PTH-Set1 (Set1) and PTH-Set1_YF/AA_ (Set1 _YF/AA_) at representative genes. ChIP-seq bedGraphs were generated by normalization to 10 million mapped reads for each sample. CRAC signals were normalized as described above. (**b**) H3K4me3, me2, me1 occupancies at the indicated genes in W303 *set1*∆::*TRP1* pRS415-nHTP-*SET1* and *SET1*_*YF/AA*_ strains. Levels of H3K4me3, me2 and me1 are normalized to total H3. Positions of the primers used for the ChIP-qPCR for each representative gene are indicated. Errors bars represent the s.d. from three independent experiments. (**c**) Recombinant chromatin template containing fully ubiquitylated H2B (H2Bub) was subjected to *in vitro* HMT assays with purified Set1C and Set1C YF/AA in the absence and presence of purified *GAL10* RNA [0.1 (lanes 2 and 7), 0.2 (lanes 3 and 8), 0.5 (lanes 4 and 9) and 2 (lanes 5 and 10) molar ratio to Set1C. H3 methylated status was monitored by western blots with indicated antibodies.

Although we showed that the Set1_YF/AA_ mutation only marginally affects the recruitment of Set1 to chromatin on a genome-wide scale, at the gene level a variegated range of cases exists. In some instances, a strong RNA-binding defect translates into a marginal effect on recruitment (for example, *MOT3*, Figure 8), in other cases (for example, *PMA1* and *ENO1*) recruitment to chromatin is affected in spite of a moderate effect on *in vivo* crosslinking to the RNA as revealed by the CRAC signal. Although it is unclear why in these latter particular cases, the Set1_YF/AA_ mutation affects Set1 occupancy, we exploited these individual differences to address the role of the nascent RNA and Set1 recruitment in H3K4 methylation. As shown in Figure 8b, in all these three cases H3K4me3 was found to be strongly reduced, indicating that neither the recruitment to chromatin (*MOT3)* nor the crosslinking to the RNA alone (*PMA1* and *ENO1*, Figure 8) are sufficient to promote methylation.

The general strong decrease in methylation when dRRM is mutated might be due to the defective positioning of the protein along transcription units, to an allosteric requirement for RNA interaction or to a general inactivation of the methylation function of Set1 by the Set1-YF/AA mutation. To distinguish between these possibilities, we analyzed *in vitro* the histone methyltransferase (HMT) activity of the Set1C containing the Set1_YF/AA_ mutation in the presence or absence of RNA. As shown in Figure 8c, Set1C YF/AA reproducibly displayed a higher HMT activity compared with WT on a recombinant chromatin template containing ubiquitylated H2B. This indicates that the Set1_YF/AA_ not only retained full HMT activity but its *in vitro* activity was even enhanced. Addition of purified *GAL1* RNA did not improve the activity of Set1C, and actually inhibited its function in a concentration-dependent manner. As expected, it had no effect when added to Set1C YF/AA (Figure 8c). This strongly suggests that the interaction with RNA is not required for activating the HMT function of Set1, and is consistent with the possibility that RNA might negatively regulate its activity.

Together, these results strongly suggest that binding to the RNA is important to define the appropriate topology of Set1C distribution along transcription units, which is important for the deposition of the H3K4me3 mark.

## Discussion

In this study, we first showed with *in vitro* studies that the interaction between Set1C and RNA is direct. Both the dRRM and N-SET domains of Set1 contribute to Set1 RNA-binding activity *in vitro* in the context of the reconstituted complex. Although structural data indicated that dRRM has the canonical structure to bind RNA [14], binding to RNA of the C762 fragment alone was unexpected because none of the C762 constituents (N-SET, SET and post-SET domains and Spp1, Bre2, Sdc1, Swd3 and Swd1) has a canonical RNA-binding motif. In addition, no direct interaction of Set1C subunits and N-SET domain alone (data not shown) with RNA was observed, strongly suggesting that interaction with RNA is mediated by a composite surface potentially involving all or some components associated with the C762 fragment. Interestingly, addition of residues 569–762 to the C762 fragment, which are known to have an inhibitory effect on Set1 methyltransferase activity [10] inhibited the RNA-binding activity of the C762 fragment, suggesting that Set1 binding to RNA could regulate the methyltranferase activity of Set1.

The involvement of the N-SET domain in RNA binding is of particular interest as this domain acts as central regulatory region of Set1C by its ability to bind Spp1 [34] and Swd1 [20]. The N-SET domain also mediates an Spp1-dependent interaction with the SET domain and its associated subunits, an interaction that likely regulates Set1 methyltransferase activity [35]. Other studies have indicated a cross-talk between Swd2 and Spp1 suggesting a complex regulation mediated by Set1C subunits for interaction between the C- and the N-terminal regions of Set1C [21]. In this work, we found that omitting Spp1 in the reconstitution assay strongly decreases Set1C RNA-binding activity. Therefore, in the context of the full-length Set1, it is possible that dRRM, Spp1 and the N-SET cooperate to stabilize interaction with RNA. *In vivo*, inhibition of the C762 RNA-binding activity by the region of Set1 encompassing amino acids 569–762 remains an open question.

We performed ChIP-seq experiments with the same Set1 mutants studied *in vitro* using an anti-Set1 mAb [24]. We provide a high-resolution map of Set1 occupancy indicating that the peak of Set1 occupancy is shifted 3′ to the peak of H3K4me3 and slightly shifted with respect to RNAPII average occupancy. Analysis of ChIP-seq signals revealed that *set1*_*YF/AA*_ mutation did not affect Set1 recruitment to chromatin per se but rather regulated Set1C distribution by maintaining Set1 in the 5′ region of genes. In addition, we show that the N-SET domain that is required to bind RNA *in vitro* is not involved in recruitment of Set1 to chromatin, whereas it is essential for Set1C catalytic activity. Assessing the functional importance of the RNA-binding activity of the N-SET remains a challenge for future studies.

To assess whether Set1 binds RNA *in vivo*, we performed CRAC experiments using Set1 and Set1_YF/AA_. Our high-resolution and strand-specific CRAC analysis shows that Set1 binds to RNA *in vivo* thereby extending and providing a physiological facet to our *in vitro* analysis with the reconstituted complex. CRAC analysis is expected to detect binding to the RNA both during and after transcription. In our experiments, the occurrence of co-transcriptional binding is demonstrated by the observation that Set1 binds to intronic regions and is also strongly suggested by the highly significant correlation with Set1 and RNAPII occupancy (as determined by ChIP and CRAC, respectively) for most features. Although it is formally possible that the levels of transcription and/or Set1 chromatin occupancy also impact to some extent the post-transcriptional binding to the RNA, we favor the hypothesis that a co-transcriptional component dominates in directing Set1 binding to the RNA for the largest fraction of the population. Binding of Set1 to the nascent transcript is not sequence specific and occurs preferentially at the 5′-end of RNAs, with a peak that is slightly shifted downstream relative to the maximum of RNAPII occupancy as detected by CRAC.

Our results indicate that the substitution of residues Y271 and F272, which are part of the hydrophobic core of RRM1 and predicted to be important for maintaining the structure of whole dRRM [36], strongly decreased Set1 RNA binding, particularly in the 5′ region of RNAs *in vivo*. This result combined with our ChIP-seq data supports the notion that Set1 is recruited via protein–protein interactions and subsequently contacts the nascent RNA 5′-region via its dRRM. Transfer of Set1C to the nascent RNA once the latter emerges from the elongation complex would contribute to position Set1 predominantly to the 5′ regions of genes.

The *set1*_*YF/AA*_ mutation markedly affects the deposition of the H3K4me3 mark at the 5′-end of genes for all genes tested consistent with the fact that global H3K4me3 is strongly reduced in the *set1*_*YF/AA*_ mutant. Importantly, the *set1*_*YF/AA*_ mutation in the context of reconstituted Set1C enhanced the methyltransferase activity of Set1C assayed on recombinant chromatin containing ubiquitylated H2B.

There might be several mechanisms by which the *set1*_*YF/AA*_ mutation affects H3K4me3. Interaction with the nascent RNA might be important to increase the persistence of Set1C in the proximity of chromatin to allow additional time for H3K4 trimethylation in the 5′-end of genes, whereas transcription proceeds at its normal speed. It is possible that binding to the RNA activates allosterically the HMT activity of the protein. However, we showed that *in vitro* addition of the RNA does not activate Set1 but actually inhibits H3K4 methylation, in a manner that depends on the RNA-binding activity of Set1. It is not clear whether this inhibition detected *in vitro* is physiologically relevant, but the possibility exists that binding to the RNA could regulate Set1 activity in some phases of the H3 methylation process. The simultaneous or sequential interaction of Set1C with the polymerase and the nascent RNA might constitute a quality control strategy to ensure deposition of the H3K4m3 mark only to regions of active transcription. Whether RNA binding to N-SET could contribute to such a process remains to be determined.

Metagene analysis experiments revealed that Set1 was also crosslinked to mRNA at the 3′-end of the molecules, showing a 3′ peak immediately before the poly(A) addition site. Importantly, formation of this 3′ RNA-binding peak was fully insensitive to the *set1*_*YF/AA*_ mutation suggesting that the binding of Set1 at poly(A) sites is independent of its dRRM. Interestingly, although the level of this peak was low relative to the levels of Set1 at the 5′-end of genes, its intensity relative to the polymerase signal (that is low in this region) is the highest over the whole transcription unit, suggesting that it might have functional significance.

The dRRM-independent formation of the 3′ Set1 peak may occur via a 3′ recruitment of Set1 by Swd2, an essential subunit of Set1C that also belongs to the APT complex, a subcomplex of the cleavage and polyadenylation factor that is involved in mRNA and snoRNA 3'-end formation [36,37]. These observations might suggest a functional link between Set1C and 3′-end formation/termination but it remains unknown how the binding of Swd2 to either of the two complexes (Set1C and APT) is regulated. Swd2 was shown to directly interact with the N-terminus of Set1 [20]. Consistent with this, Swd2 recruitment to the 5′ region of genes is reduced when *SET1* is deleted [38] but whether Swd2 contributes to recruit Set1C at the vicinity of the poly(A) site to signal cleavage and polyadenylation remains to be determined.

We also observed a striking enrichment of Set1 to snRNA, suggesting that Set1 could fulfill a function in signaling splicing events. Consistent with this notion we observed that Set1 is enriched within introns, even when the signal is evaluated relative to the RNA polymerase (that also increases in introns).

Interestingly, cryptic unstable transcripts were under-represented when compared with matched size small mRNAs, opening the possibility that at early stages of ncRNA (non-coding RNA) transcription, dRRM may compete with Nab3 RRM (of the Nrd1 complex) for the recognition of sequences in the nascent transcript. This might explain the more efficient termination of ncRNA observed in cells lacking Set1 [39,40].

Finally, we show that Set1 binds to a class of transcripts to an extent that cannot be justified by a co-transcriptional component alone, or, at least, not by the same co-transcriptional component that holds for the majority of the population. We therefore suggest that Set1 can bind RNAs after transcription or that binding occurs during transcription but additionally persists in this class of genes. The very high level of Set1 bound to *SET1* mRNA was previously uncovered [26] and probably reflects the co-translational assembly mode of Set1C. In this model, *SET1* mRNA is bound by the nascent Set1 protein that emerges from the ribosome through indirect interactions with the translation machinery.

Set1 also binds post-transcriptionally to other transcripts. Interestingly, many of these factors are transcription factors some of which are functionally related. For instance, Mot3 and Rox1, which are transcriptional repressors of genes encoding cell wall proteins [36] and of hypoxic genes [37] are functionally linked with Msn2-Msn4 in the osmostress response [36] and with Sok2 in the ergosterol biosynthetic pathway [41]. Interestingly, Set1 was previously described to be required for the expression of genes in the ergosterol biosynthetic pathway [42]. Strikingly, Set1 binds also to mRNA of several genes functionally related to chromosome segregation, in line with recent results linking Set1 to mitotic spindle assembly [43,44]. However, the steady-state levels of these transcripts were not affected by deletion of *SET1*, at least in rich medium (YPD) [39], suggesting that Set1 might affect the expression of these genes at levels that do not involve mRNA synthesis or degradation rates, or that the impact of Set1 is revealed only under defined growth or stress conditions.

Our results also show that Set1 binds to Ty1 mRNA and repress Ty1 mobility at a post-transcriptional stage. Our data uncover a new function of Set1C as a repressor of Ty1 mobility and add another layer of regulation by Set1 to previous studies showing that Set1 had a synergistic role with the histone H4 metyhyltransferase Set5 in repressing transcription of Ty transposable elements [23]. Although Ty1 retrotransposition can alter yeast genome integrity and is consequently a highly controlled process, release of Ty1 repression is supposed to contribute to genome evolution and cell adaptation to stress [45]. Therefore, it would be interesting to determine whether Set1C repression could be alleviated under stress conditions that are known to stimulate Ty1 retrotransposition [46,47].

Deciphering the role of the post-transcriptional binding of Set1 to RNAs will reveal unexpected function of Set1C that might explain the incredibly complex genetic interaction map of Set1 [48].

## Materials and methods

### Purification of recombinant Set1C and subunits, *in vitro *RNA electrophoretic mobility shift assay, and *in vitro* HMT assay

Preparation of FLAG-tagged recombinant Set1C and subunits were as described [20]. For radiolabeled RNA probe preparation, DNA duplexes containing T7 promoter sequence ( 5′-TAATACGACTCACTATAGGG-3′) followed by 100-nucleotide sequence encoding *GAL1* or *GAL10* mRNA starting from the transcription start site were generated by PCR. *GAL1* and *GAL10* RNA were transcribed by T7 RNA polymerase according to the manufacturer’s instructions (Promega, Fitchburg, WI, USA) and then purified by gel elution method. After removing 5′ phosphate by Antarctic phosphatase, the 5′-end of RNA was radiolabeled by T4 polynucleotide kinase using [γ-32P]ATP and then purified by Sephadex column (iNtRon, Seongnam, South Korea) and gel elution method. For *in vitro* RNA electrophoretic mobility shift assay, reactions containing purified Set1C or individual subunits and 0.25 pmole of radiolabeled RNA in 20 μl reaction buffer (10 mM Tris-Cl (pH 7.5), 1 mM EDTA, 5 mM MgCl_2_, 50 mM K-glutamate, 5% glycerol, 1 mM DTT and two units of RNasin) were incubated at room temperature for 30 min. The samples were resolved by electrophoresis at 4 °C on 5% polyacrylamide gels in 1×TBE buffer and subjected to autoradiography. For *in vitro* HMT assay, 40 μl reactions containing 350 ng (histone amount) recombinant chromatin assembled as described in Kim *et al.* (2013) with H2Bub-containing histone octamer [49], purified Set1C and 100 μM S-adenosylmethionine were incubated at 30 °C for 2 h. Proteins were resolved by sodium dodecyl sulfate–polyacrylamide gel electrophoresis and subjected to western blots.

### Strains, constructs and growth conditions

For reconstitution of Set1Cs, *SET1, set1* mutants and Set1C subunits genes were subcloned in pFASTBAC1 with or without a FLAG tag [20]. Baculoviruses were generated according to the manufacturer’s instruction (Gibco-Invitrogen, Waltham, MA, USA). Sf9 cells were infected with combinations of baculoviruses and proteins/complexes were affinity purified on M2 agarose (Sigma, St Louis, MO, USA) as described [20].

Yeast strains and primers used in this study are described in Supplementary Tables S2 and S3, respectively (see Supplementary Information). Full-length *SET1* and *SET1* mutants were cloned into in pRS415-nHTP [23]. Expression of the resulting constructs (*Z-tag*—*TEV cleavage site*—*His6*—*SET1*) is under the control of *MET25* promoter. The pRS415-nHTP-*SET1* (or *SET1* mutants) were transformed into W303 *set1∆*::*TRP1* strain. Plasmid pRS415-nHTP-*SET1* complements all the tested *set1∆-*associated phenotypes of the *set1∆::TRP1* strain. For ChIP-seq and CRAC experiments, W303 *set1∆*::*TRP1* pRS415-nHTP-*SET1* (or *SET1* mutants) cells were grown in SC-TRP-LEU. RNAPII CRAC experiments were performed from W303 cells expressing Rpb1-HTP and grown in SC-TRP. Construction of fully functional chromosomally encoded Myc9-tagged Set1 is described in Dehe *et al*. [10].

### CRAC analyses

Cells in exponential phase were crosslinked with a Megatron for 100 s (Set1) and 50 s (Rpb1), harvested by centrifugation, resuspended in 2.4 volume/g of cells of TN150 buffer (50 mM Tris pH 7.8, 150 mM NaCl, 0.1% NP-40 and 5 mM beta mercaptoethanol) supplemented with protease inhibitors (complete, Mini, EDTA-free Protease Inhibitor Cocktail). This suspension was flash frozen in droplets and cells were mechanically broken using the Mixer Mill MM 400 by doing five cycles of 3 min at 20 Hz. A non-crosslinked sample was treated in parallel as a background control.

Powders were thawed and the resulting extracts were treated for one hour at 25 °C with DNase I (165 U/g of cells) to solubilize chromatin and then clarified by centrifugation for 20′ at 20 000 *g* at 4 °C. Subsequent purifications steps were performed essentially as described with minor modifications from Granneman *et al.* [50]. For both nPTH-Set1 and Rpb1-HTP strains, adaptors were modified in order to sequence RNA molecules from the 3′-end.

The RNA was recovered after proteinase K treatment and reverse transcribed using specific primers. The resulting complementary DNA was used to perform multiple PCR reactions in a final volume of 25 μl using the following conditions: 0.4 μM of each primers 0.2 mM dNTP, 2.5 U LA Taq DNA polymerase from Takara, 1X LA PCR Buffer II and 2 μl of complementary DNA per reaction with the programme: 2′ at 95 °C, (30′′ at 95 °C, 45′′ at 58 °C, 1′ at 72 °C) × 13 cycles, 5′ at 72 °C. PCR were pooled and treated with 200 U of Exonuclease I (NEB) per milliliter of PCR reaction for 1 h at 37 °C. After Exonuclease I inactivation for 20′ at 80 °C, DNA was purified on PCR clean up columns (NucleoSpin Gel and PCR Clean-up, Macherey-Nagel, Düren, Germany) and sequenced using Illumina technology (San Diego, CA, USA). Primers are indicated in Supplementary Table S2.

Samples were demultiplexed using the pyBarcodeFilter script from the pyCRAC utility suite. Subsequently, the 3′ adaptor is clipped with Cutadapt and the resulting insert is quality trimmed from the 3′-end using Trimmomatic rolling mean clipping (window size=5, minimum quality=25). At this stage, the pyCRAC script pyFastqDuplicateRemover is used to collapse PCR duplicates and ensure each insert is represented only once. Each unique insert in our library is associated with a six-nucleotides random tag within the 5′ adaptor. The resulting sequences are reverse complemented with Fastx_reverse_complement (part of the fastx toolkit [51]), and mapped to the R64 genome (sgd) with bowtie2 (-N 1 –f).

Read counts were normalized relative to reads derived from an *S. pombe* spike that was added to *S. cerevisiae* cells before the crosslinking step. The *S. pombe* spike cells contain a non-relevant protein tagged with the same HTP tag that was co-purified with the *S. cerevisiae* material. The positionally weighted average poly(A) addition site (wPAS) for every gene was calculated by weighting the position of each poly(A) site using its intensity and calculating an average position.

### ChIP-seq, data processing and ChIP-qPCR

ChIP of Myc-Set1 and PTH-Set1 were performed as previously described [52] with 9E10 (anti-MYC, Santa Cruz Biotechnology, Dallas, TX, USA) and anti-Set1 monoclonal antibodies (P Nagy, University of Toronto, Toronto, Canada). Libraries were prepared from fragmented DNA using the Chip-seq MicroPlex Library Preparation Kit v2 samples preparation (Diagenode, Seraing, Belgium) according to the manufacturer’s instructions. In all, 2 ng from IP samples were used as the starting material. Each library was barcoded using MicroPlex Single Index (Diagenode): iPCRtagT5, T6, T7 and T8 and amplified for 10 and 6 cycles for IP and input samples, respectively. Each library was quantified on Qubit with Qubit dsDNA HS Assay Kit (Life Technologies, Carlsbad, CA, USA) and then, size distribution was examined on the Bioanalyser with High Sensitivity DNA chip (Agilent, Santa Clara, CA, USA), to ensure that the samples have the proper size, no adaptor contamination and to estimate sample molarity. Each library was diluted to 4 nM and then pulled together at equimolar ratio. Libraries were denatured according to the manufacturer’s instruction and sequenced on a mid-output flow cell (130 M clusters) using the NextSeq 500/550 High Output v2 150 cycles kit (Illumina), in paired-end 75/7 nt mode, according to the manufacturer’s instructions. In all, 148 million (M) paired-end reads were generated (34–39 M per sample) with 93% >= Q30.

ChIP-Seq data quality was assessed using FastQC. FasQC: a quality control tool for high-throughput sequence data. Available online at: http://www.bioinformatics.babraham.ac.uk/projects/fastqc. Sequencing reads (FastQ format) were mapped to the *Saccharomyces cerevisiae* genome (sacCer3) using BFAST alignment tool with default parameters [53] (PMID 19907642) to obtain a Binary Alignment Mapped (BAM) file. The sorted BAM files were used to determine average profiles of ChIP-Seq read density using *ngs.plot* software [54], (PMID 24735413) around the transcription start site. Read counts were normalized to the total number of million uniquely mapped reads or to read count per million of mapped reads (RPM). The RPM values allow samples to be compared regardless of differences in sequencing depth. To generate BedGraphs for visualization on genome browsers, ChIP-Seq BAM files were processed using HOMER package. The tag directory for each sample was then created using the makeTagDirectory tool and the corresponding BedGraph was generated using makeUCSCfile tool with default options. Only uniquely mappable reads (non-secondary alignment) were considered to create BedGraphs with a normalization to 10 million mapped reads for each sample. To compare Set1 binding positions on RNA with Set1 occupancy on genes of interest, the Multicov command from Bedtools [55] was used to obtain read counts within each gene.

For ChIP-qPCR, samples were prepared as previously described [52]. DNA was analyzed by real-time qPCR using SYBR Green Premix Ex Taq (Takara, Mountain View, CA, USA) in a Rotor Gene 6000 (Corbett Research, Labgene, Archamps, France). Primers are listed in Supplementary Table S2. The following antibodies were used: anti-H3 (Abcam1791, Cambridge, UK), anti-H3K4me2 (Abcam-ab7766, Cambridge, UK), anti-H3K4me3 (Abcam-ab8580), anti-Myc 9E10 (Santa Cruz Biotechnology-sc-40) and anti-Rap1 (V. Géli’s laboratory, Marseille, France).

### Data access

ChIP-seq data sets (PTH-Set1, PTH-Set1_YF/AA_, PTH-Set1_∆dRRM,_ PTH-Set1_∆NSET,_ PTH-Set1_∆RRM_ ∆NSET, PTH-vector, Input PTH-Set1 and input PTH-Set1_YF/AA_), as well as CRAC sequences generated during this work are deposited to the NCBI Gene Expression Omnibus (GEO; http://www.ncbi.nlm.nih.gov/geo/) under the accession number GSE104486 (GSE104484 for chiPseq datasets and GSE104485 for CRAC datasets).

### Ty1 transposition assays

The pOY1 *URA3*, centromeric vector carrying a Ty1-*his3AI* reporter element expressed from its own promoter was previously described [33]. Total RNAs were extracted from yeast cultures after 4 h or 8 h at 20 °C, for Ty1 mRNAs or Ty1-*his3AI* mRNAs respectively, using the Nucleospin RNA II kit (Macherey-Nagel) and reverse transcribed with Superscript-II reverse transcriptase (Invitrogen, Waltham, MA, USA). Complementary DNA quantification was achieved by real-time PCR with a LightCycler 480 system (Roche, Basel, Switzerland) using SYBR Green incorporation according to the manufacturer’s instructions. The amounts of the mRNAs of interest were normalized relative to 25S ribosomal RNA values. Primers used are described in Supplementary Table S2.

To estimate the frequency of Ty1*his3AI* mobility [32], overnight liquid cultures were grown at 30 °C from an individual clone in HC medium (Hartwell’s synthetic complete) [56], lacking uracil and supplemented with 2% glucose. Each culture was diluted to OD 0.01 in HC-URA medium and grown to saturation at 20 °C, which is permissive for Ty1 transposition. In all, 3 ml of each culture were plated on two HC agar plates lacking histidine. Cell titer was determined by plating 10 000-fold diluted cultures on YEPD rich medium. Plates were incubated for 3 days at 30 °C and counted to determine the fraction of [HIS^+^] prototrophs. Ty1 retrotransposition frequencies were defined as the mean of 3 experiments, each one performed with four independent clones.
