# Supplementary material for: Binding to RNA regulates Set1 function
Source: Cell Discov. 2017 Oct 24;3:17040–. doi: 10.1038/celldisc.2017.40 (PMC5654745; doi:10.1038/celldisc.2017.40)
Supplement: Supplementary Information [file celldisc201740-s1.pdf]

Binding to RNA regulates Set1 function

Pierre Luciano[1,5], Jongcheol Jeon[2,5], Abdessamad El-kaoutari[1,5], Drice Challal[3,5], Amandine Bonnet[4], Mara Barucco[3], Tito Candelli[3], Frederic Jourquin[1], Pascale Lesage[4], Jaehoon Kim[2,*], Domenico Libri[3,*], Vincent Géli[1,*].

Supplementary information

Supplementary Figure S1. *In vitro* interaction of RNA with individual subunits of Set1C

(**A**) SDS-PAGE and Coomassie blue staining of purified Set1C subunits.

(**B**) Radiolabeled *GAL10* transcripts were subjected to *in vitro* RNA electrophoretic mobility shift assay with purified Set1C subunits. 0.5 pmoles (lanes 4, 6, 8, 12, 14, 16) or 2.5 pmoles (lanes 5, 7, 9, 11, 13, 15, 17) of each subunit were added. Set1C is shown as a positive control at the same concentrations.

Supplementary Figure S2. Expression of the PTH-Set1 and characterization of the anti-Set1 mAb

(**A**) Set1 amount in W303 and in W303 *set1Δ::TRP1* pRS415-Z-tag-Tev-6His-*SET1* (PTH-Set1) cells grown in SC -TRP-LEU-MET versus SC-TRP-LEU. Set1 and PTH-Set1 are detected with anti-Set1 mAb.

(**B and C**) The anti-Set1 mAb recognized an epitope comprised in a Set1 region lying between residue 700 and 761. **(B)** Reconstituted Set1C- containing either Flag-Set1 (Full length, FL) or the indicated Flag-Set1 truncations- were analysed by Western blot either with anti-Flag (top) or anti-Set1 mAb (bottom). **(C)** Yeast strains expressing the indicated Set1 deletion mutants (Soares et al. 2014) were analysed by Western blot with anti-Set1 mAb.

Only the Set1 mutant lacking the region from 700 to 761 is not recognized by the anti-Set1 mAb.

Supplementary Figure S3. Comparison of Myc-Set1 and PTH-Set1 occupancy profiles

(A) Enrichment profiles of Myc-Set1 and PTH-Set1 at the indicated region of Chr VII. Graphs are normalized to 10 million mapped reads for each ChIP-seq.

(B) Occupancy snapshots of Myc-Set1 and PTH-Set1 on representative genes.

(C) The correlation plot between Myc-Set1 and PTH-Set1 datasets. Myc-Set1 and PTH-Set1 read coverages were compared by Pearson correlation. Read coverages were computed using deepTools utility multiBamSummary version 2.5.3 after binning in 100 bp intervals and calculating the count per regions.

(**D**) Metagene analysis of PTH-Set1 occupancy on big genes (> 1500 bp). Enrichment profiles were compared to those of H3K4me1, me2, and me3 [50] and RNAPII [57].

Supplementary Figure S4. Distribution of ChIP signals for PTH-Set1 and PTH-Set1$_{YF/AA}$ in the whole population of mRNA coding genes

Supplementary Figure S5. Distribution of Set1 CRAC reads across transcript classes. Reads for rRNA have not been included as they are strongly represented in the sequencing reaction from the non-crosslinked sample and it cannot be established if these represent artefacts or bona fide signals.

Supplementary Figure S6. Specific classes of mRNA are highly bound by Set1 relative to RNAPII

(A) Examples of transcripts with high Set1/RNAPII CRAC signals. The RNAPII CRAC (Blue) and Set1 CRAC (green) signals are shown. The scale is indicated for each snapshot.

(B) BAP2 illustrates a transcript that is co-transcriptionally bound by Set1.

(C) Integration of protein-protein interactions among genes whose mRNA are highly bound by Set1, including direct (physical) as well as indirect (functional) associations. The graph was performed using the STRING database.

Supplementary Table Legends.

Supplementary Table S1. CRAC and ChIP-seq datasets.

Reads are indicated. Set1CL: Set1 cross-linked; Set1noCL:Set1 not Cross-linked.

Supplementary Table S2. Strains used in this study

Name	genotype	Ref.
W303-1A	Mat a leu2-3,112 trp1-1 can1-100 ura3-1 ade2-1 his3-11,15	Rothstein RJ (1983)
W303-1A Myc-Set1	Mat a leu2-3,112 trp1-1 can1-100 ura3-1 ade2-1 his3-11,15 Myc-Set1::TRP1	Dehé et al. 2016
W303-1A *set1::TRP1*	Mat a leu2-3,112 trp1-1 can1-100 ura3-1 ade2-1 his3-11,15 *set1::TRP1*	This study
W303-1A *set1::TRP1* pRS415-nHTP	Mat a leu2-3,112 trp1-1 can1-100 ura3-1 ade2-1 his3-11,15 *set1::TRP1* pRS415-nHTP	This study
W303-1A *set1::TRP1* pRS415-nHTP-*SET1*	Mat a leu2-3,112 trp1-1 can1-100 ura3-1 ade2-1 his3-11,15 set1Δ::TRP1 pRS415-nHTP-SET1	This study
W303-1A *set1::TRP1* *pRS415-nHTP-*$set1_{YF/AA}$	Mat a leu2-3,112 trp1-1 can1-100 ura3-1 ade2-1 his3-11,15 set1::TRP1 *pRS415-nHTP-* $set1_{YF/AA}$	This study
W303-1A *set1::TRP1* *pRS415-nHTP-* $set1_{\Delta RRM}$	Mat a leu2-3,112 trp1-1 can1-100 ura3-1 ade2-1 his3-11,15 set1::TRP1 *pRS415-nHTP-* $set1_{\Delta RRM}$	This study
W303-1A *set1::TRP1* *pRS415-nHTP-* $set1_{\Delta N\text{-}SET}$	Mat a leu2-3,112 trp1-1 can1-100 ura3-1 ade2-1 his3-11,15 *set1::TRP1* *pRS415-nHTP-* $set1_{\Delta N\text{-}SET}$	This study
W303-1A *set1::TRP1* *pRS415-nHTP-* $set1_{\Delta RRM, \Delta N\text{-}SET}$	Mat a leu2-3,112 trp1-1 can1-100 ura3-1 ade2-1 his3-11,15 *set1::TRP1* *pRS415-nHTP-* $set1_{\Delta RRM, \Delta N\text{-}SET}$	This study
W303-1A *set1YF/AA::TRP1*	Mat a leu2-3,112 trp1-1 can1-100 ura3-1 ade2-1 his3-11,15 *set1YF/AA::TRP1*	This study

Supplementary Table S3. Primers used in this study

Name	Sequence 5' to 3'	
5’-PMA1-F	TCAGCTCATCAGCCAACTCAAG	qPCR
5’-PMA1-R	CGTCGACACCGTGATTAGATTG	
3’-PMA1-F	TACTGTCGTCCGTGTCTGGATCT	
3’-PMA1-R	CCTTCATTGGCTTACCGTTCA	
5'-MOT3-F	AACACGACTACTGTTTCCTCT	
5'-MOT3-R	AAGGGTATATATACTGCTGCT	
3'-MOT3-F	GTTACGATACAAACATCAAGA	
3'-MOT3-R	CTATTTGTTGTGACTAACAAT	
5'-ENO1-F	CGATGACTTCTTGATTTCTTT	
5'-ENO1-R	GTGCTTGTATAATGGGACATT	
3'-ENO1-F	ACTTTCATTGCTGACTTGGTC	
3'-ENO1-R	AACAGCGTTGTCACCTAATTC	
5'-Ty1	CATTGCGTCAAATGAGATCCAA	
3'-Ty1	GGTGTGGAATCGGTTGGACTC	
5'-Ty1-HIS3	TGTGATGACAAAACCTCTTCCG	
3'-Ty1-HIS3	ACGATGTTCCCTCCACCAAA	
5'-25S rRNA	AACGTCTATGCGAGTGTTTGG	
3'-25S rRNA	TTCCTCTGGCTTCACCCTATT	
L3-6N-GA	/5rApp/GCTtcNNNNNNAGATCGGAAGAGCGTCGTGTAGGGAAAG AGTGT/3ddC/	3' adapter
L3-6N-GU	/5rApp/GCTacNNNNNNAGATCGGAAGAGCGTCGTGTAGGGAAAG AGTGT/3ddC/	
L3-6N-AC	/5rApp/GCTgtNNNNNNAGATCGGAAGAGCGTCGTGTAGGGAAAG AGTGT/3ddC/	
L3-6N-UC	/5rApp/GCTgaNNNNNNAGATCGGAAGAGCGTCGTGTAGGGAAAG AGTGT/3ddC/	
L5miRCat	5-/5InvddT/CTTGrGrCrArCrCrCrGrArGrArArUrUrCrCrA-3	5' adapter
RT L3-2	ACACTCTTTCCCTACACGACGCTCTTCCG-3	RT
P5_3prime	AATGATACGGCGACCACCGAGATCTACACTCTTTCCCTACACGA CGCTCTTCCGATCT	PCR
miRCat_PCR2	CAAGCAGAAGACGGCATACGAgatcCTTGGCACCCGAGAAT	PCR

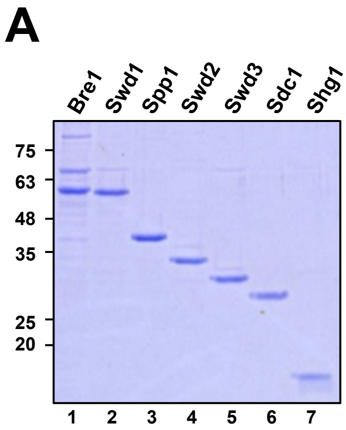
A
Bre1
Swd1
Spp1
Swd2
Swd3
Sdc1
Shg1
75
63
48
35
25
20
1
2
3
4
5
6
7

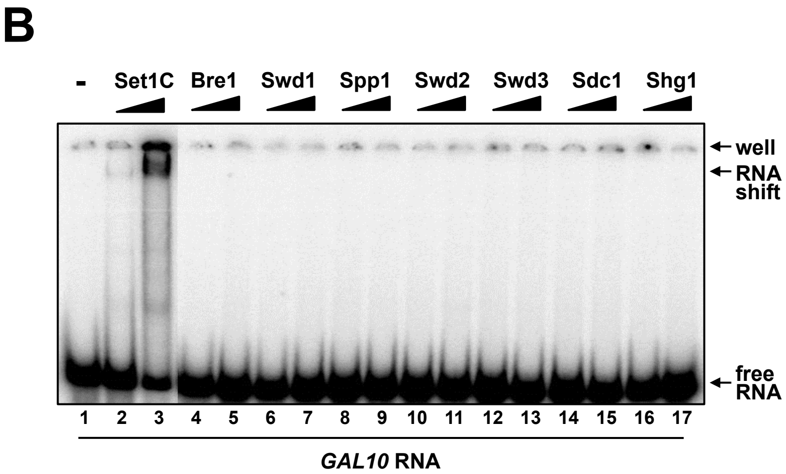
B
-
Set1C
Bre1
Swd1
Spp1
Swd2
Swd3
Sdc1
Shg1
well
RNA shift
free RNA
1
2
3
4
5
6
7
8
9
10
11
12
13
14
15
16
17
GAL10 RNA

Figure S1

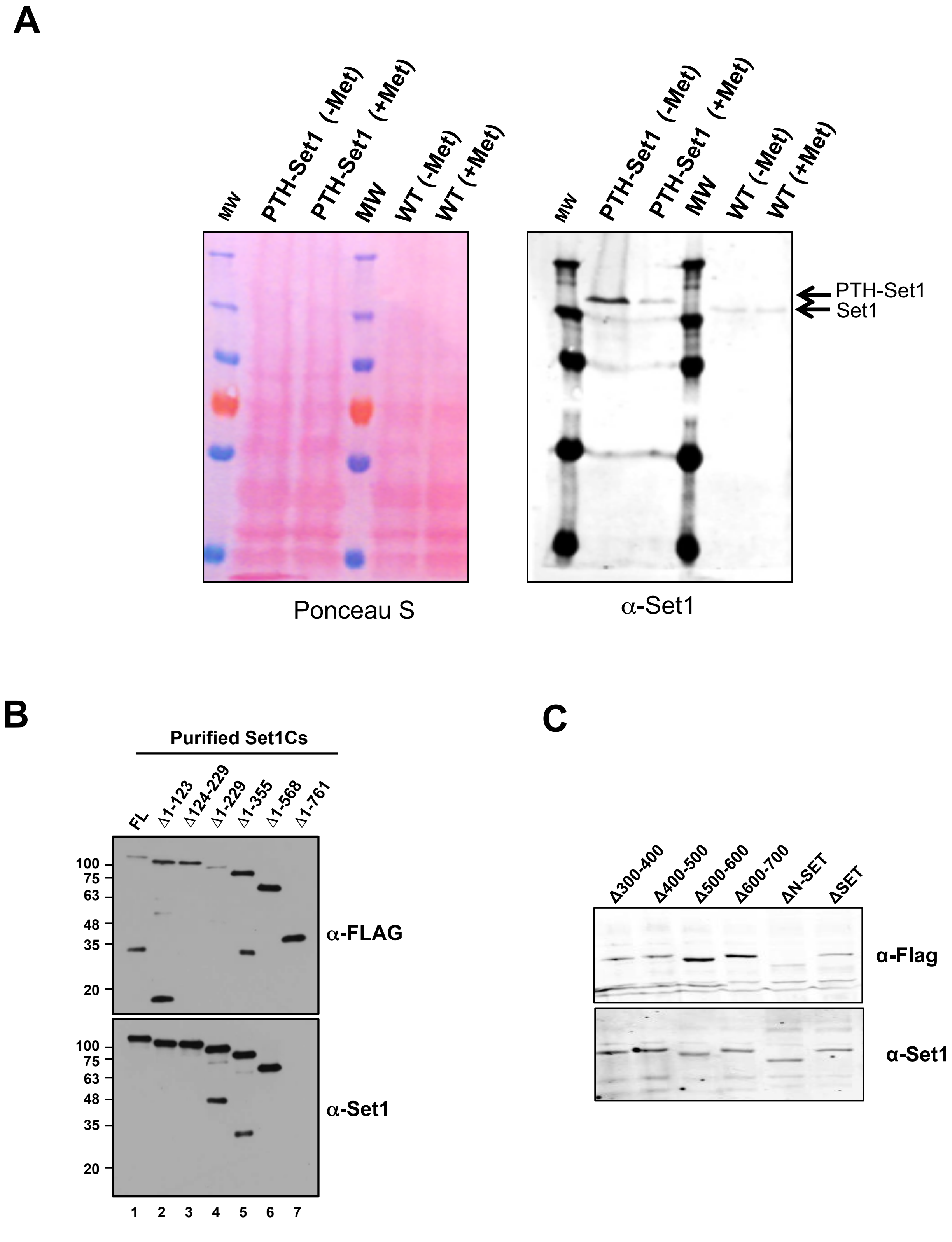
A
MW
PTH-Set1 (-Met)
PTH-Set1 (+Met)
MW
WT (-Met)
WT (+Met)
Ponceau S
MW
PTH-Set1 (-Met)
PTH-Set1 (+Met)
MW
WT (-Met)
WT (+Met)
PTH-Set1
Set1
α-Set1
B
Purified Set1Cs
FL
Δ1-123
Δ124-229
Δ1-229
Δ1-355
Δ1-568
Δ1-761
100
75
63
48
35
20
α-FLAG
100
75
63
48
35
20
α-Set1
1
2
3
4
5
6
7
C
Δ300-400
Δ400-500
Δ500-600
Δ600-700
ΔN-SET
ΔSET
α-Flag
α-Set1

Figure S2

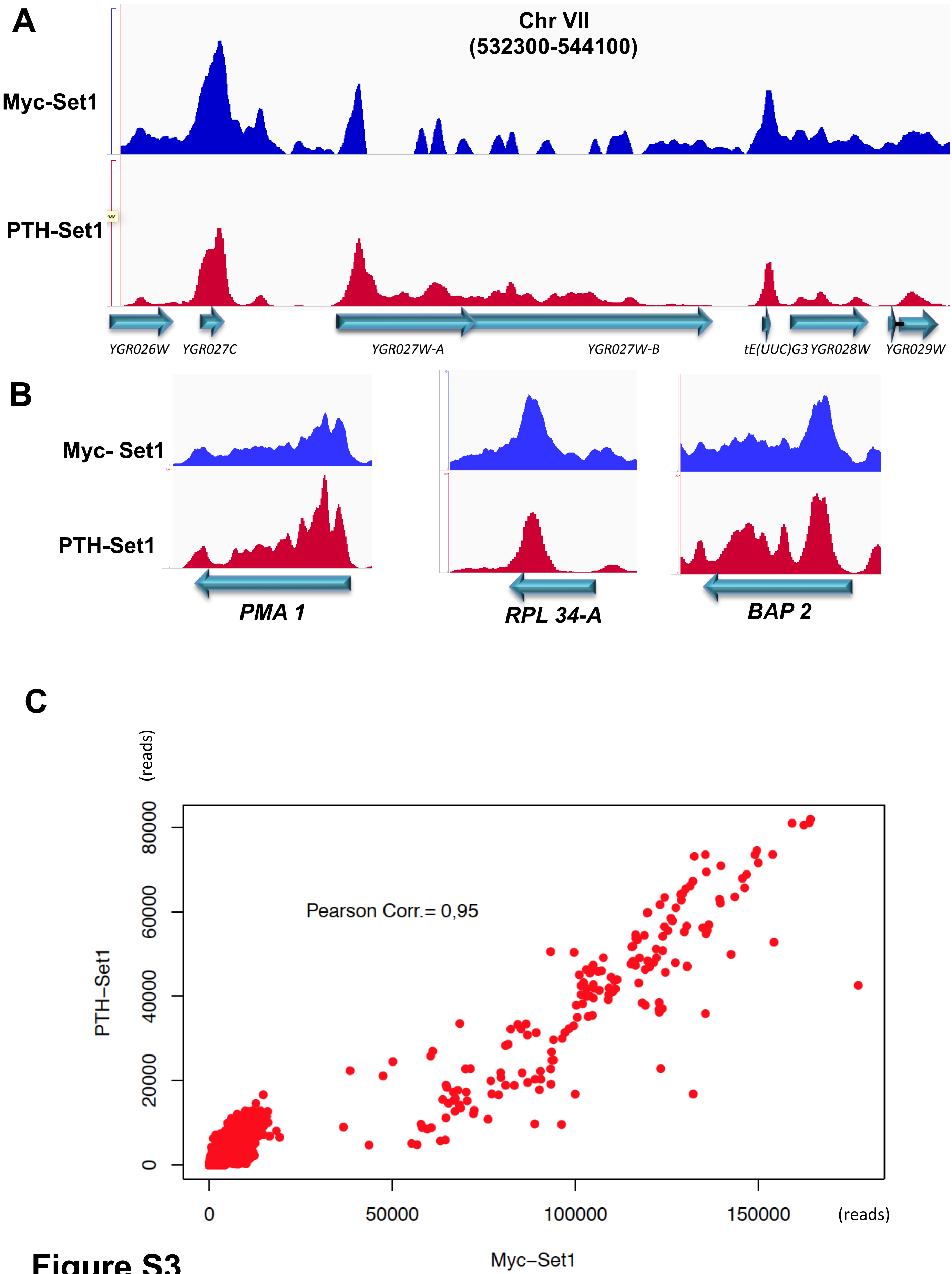
A
Chr VII
(532300-544100)
Myc-Set1
PTH-Set1
YGR026W
YGR027C
YGR027W-A
YGR027W-B
tE(UUC)G3
YGR028W
YGR029W
B
Myc- Set1
PTH-Set1
PMA 1
RPL 34-A
BAP 2
C
Pearson Corr.= 0,95
PTH–Set1
(reads)
0
20000
40000
60000
80000
0
50000
100000
150000
(reads)
Myc–Set1

Figure S3

D

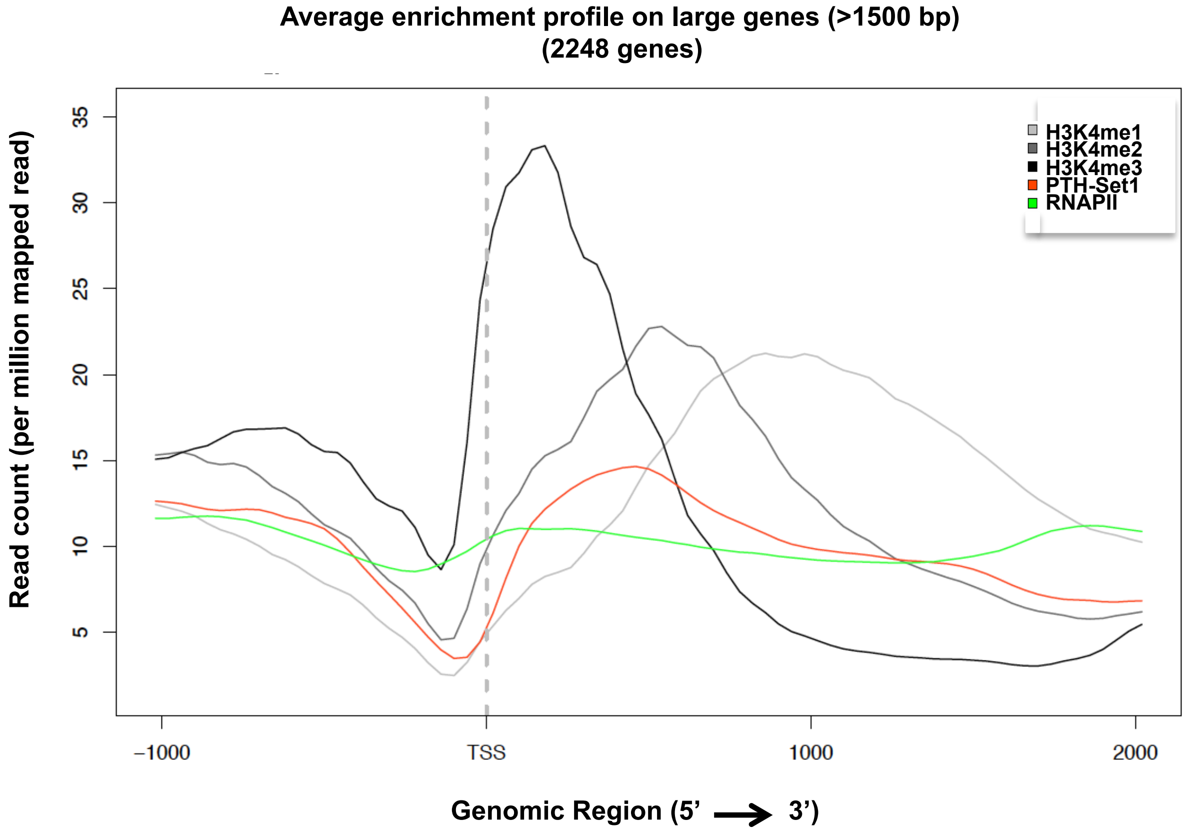
Average enrichment profile on large genes (>1500 bp)
(2248 genes)
Read count (per million mapped read)
35
30
25
20
15
10
5
H3K4me1
H3K4me2
H3K4me3
PTH-Set1
RNAPII
−1000
TSS
1000
2000
Genomic Region (5' → 3')

Figure S3

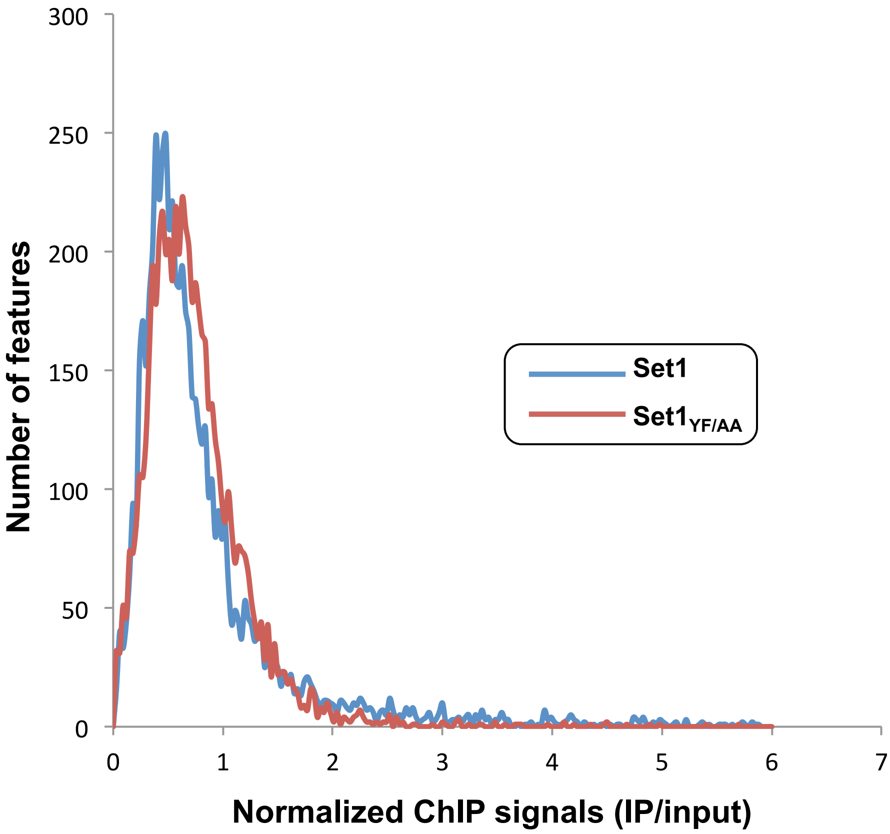
300
250
200
150
100
50
0
Number of features
0
1
2
3
4
5
6
7
Normalized ChIP signals (IP/input)
Set1
Set1YF/AA

Figure S4

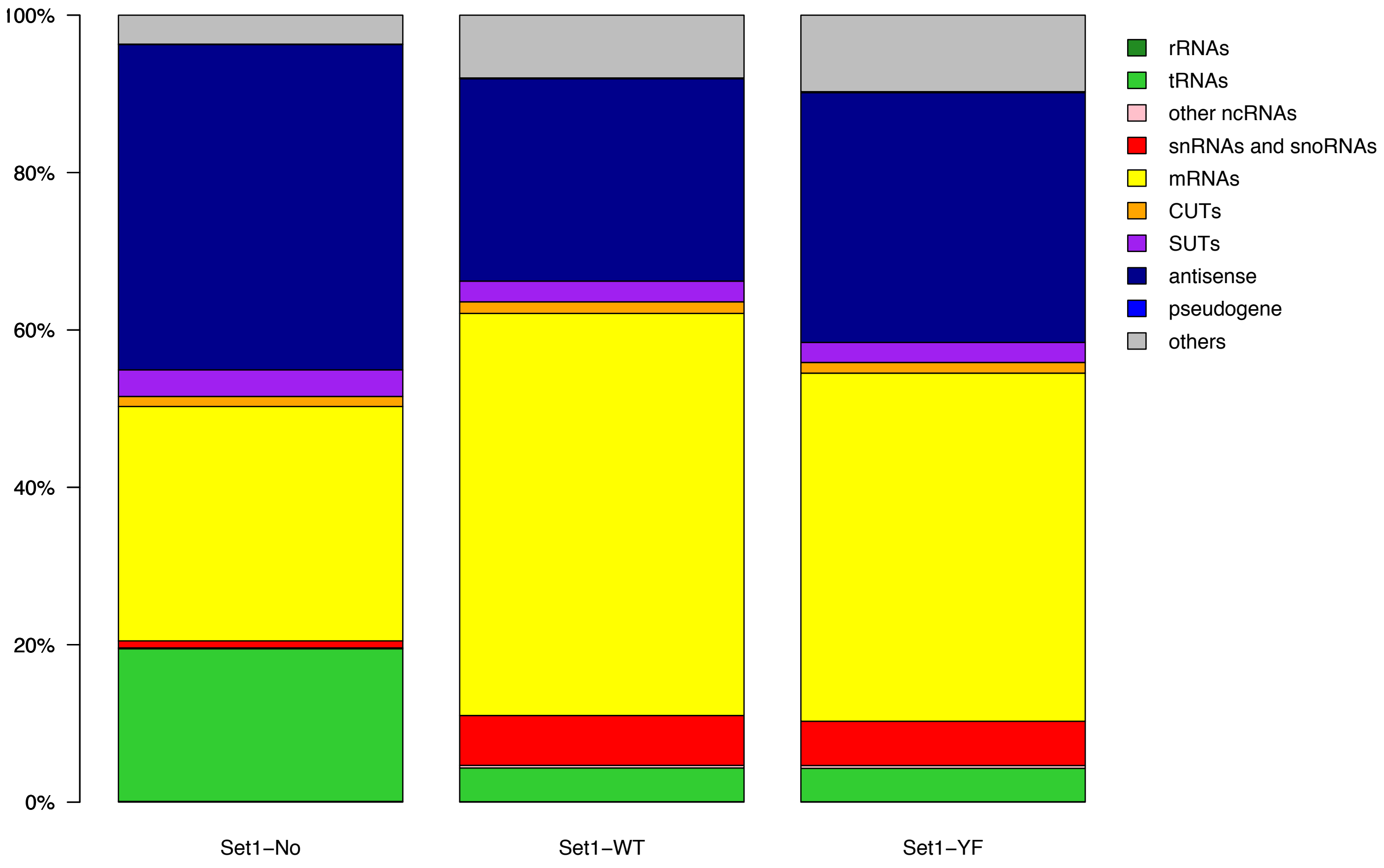
100%
80%
60%
40%
20%
0%
Set1−No
Set1−WT
Set1−YF
rRNAs
tRNAs
other ncRNAs
snRNAs and snoRNAs
mRNAs
CUTs
SUTs
antisense
pseudogene
others

Figure S5

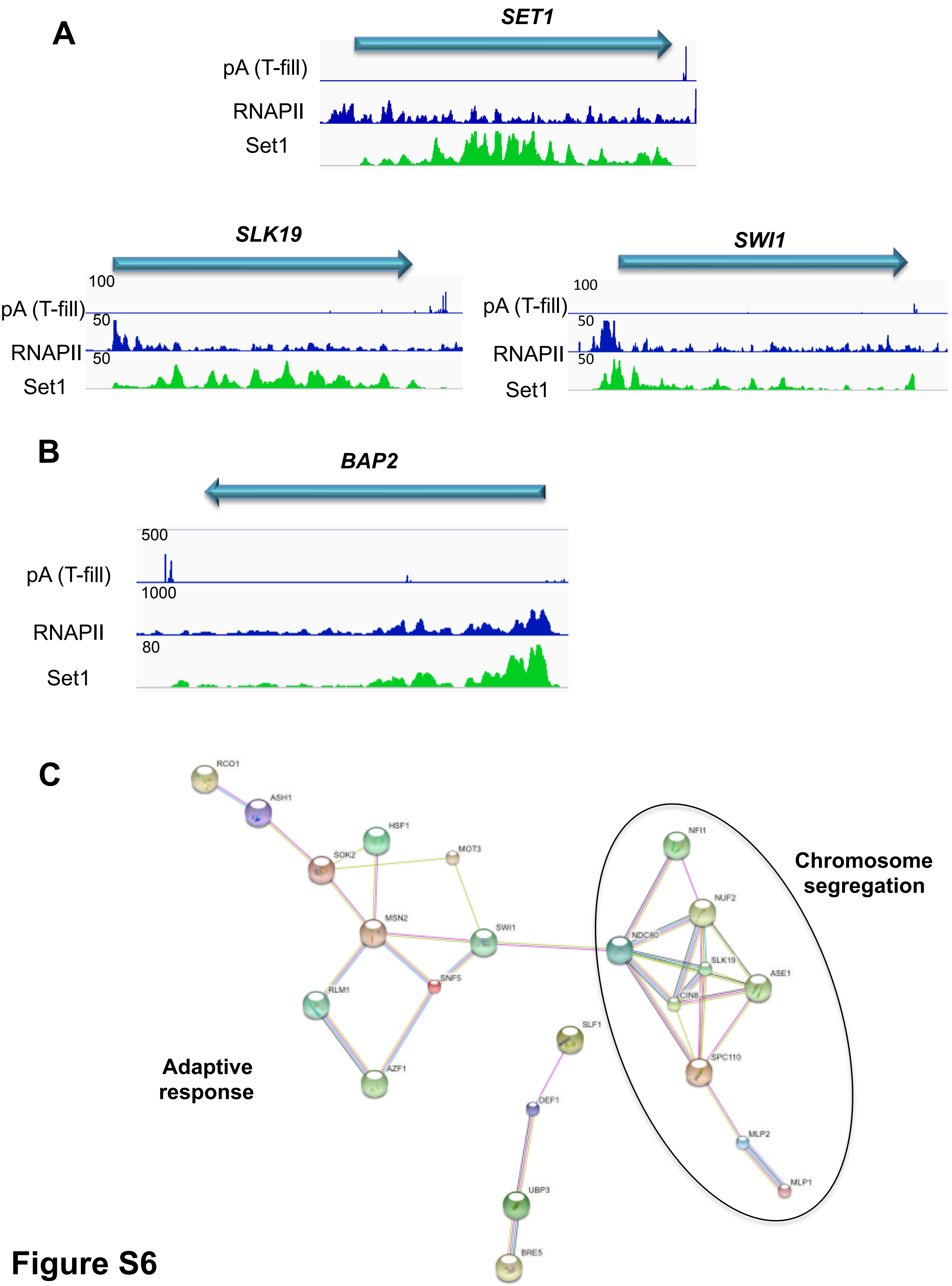
A
SET1
pA (T-fill)
RNAPII
Set1
SLK19
100
pA (T-fill)
50
RNAPII
50
Set1
SWI1
100
pA (T-fill)
50
RNAPII
50
Set1
B
BAP2
500
pA (T-fill)
1000
RNAPII
80
Set1
C
RCO1
ASH1
HSF1
SOK2
MOT3
MSN2
SWI1
SNF5
RLM1
AZF1
Adaptive response
NFI1
NUF2
NDC80
SLK19
ASE1
CIN8
SPC110
MLP2
MLP1
Chromosome segregation
SLF1
DEF1
UBP3
BRE5

Figure S6
